# Perinatal mental health and reproductive behavior among women in wartime Ukraine: a cohort study protocol

**DOI:** 10.3389/fpsyt.2026.1874060

**Published:** 2026-07-06

**Authors:** Liudmyla Krupelnytska, Nazar Yatsenko, Antonina Molotokas, Olha Morozova-Larina, Alona Vavilova, Artem Baratiuk, Stanislav Kazakov, Volodymyr Artyomenko

**Affiliations:** 1 Department of Psychodiagnostics and Clinical Psychology, Taras Shevchenko National University of Kyiv, Kyiv, Ukraine; 2 Scientific School “Clinical Psychology”, Taras Shevchenko National University of Kyiv, Kyiv, Ukraine; 3 Department of Obstetrics and Gynecology, Odesa National Medical University, Odesa, Ukraine; 4 Obstetrical and Labor Department, Odesa City Maternity Hospital No. 5, Odesa, Ukraine

**Keywords:** cohort study, perinatal mental health, postpartum, pregnancy, reproductive behavior, Ukraine, war-affected population, women

## Abstract

**Background:**

War reshapes the conditions under which reproductive decisions are made and pregnancy is experienced. Prolonged insecurity, displacement, economic instability, disruption of healthcare, and reduced social support may lead women and families to postpone pregnancy or face pregnancy and childbirth under constrained conditions. These challenges highlight the need for research on how war-related stress and trauma affect women’s reproductive behavior and perinatal mental health.

**Method:**

This prospective cohort study will recruit at least 328 women through healthcare institutions providing reproductive and perinatal care. Eligible participants will include non-pregnant women, pregnant women, and women within the first year postpartum. Where feasible, partners of enrolled women will also be included to provide complementary dyadic data. Participants will be followed from baseline to 3, 6, and 12 months. Data will be collected through web-based questionnaires and routine clinical records. No experimental intervention will be delivered, and participation in the study will not modify standard clinical care. Repeated measures will capture mental health, reproductive intentions and behavior, war-related and perinatal experiences, and key sociodemographic and obstetric characteristics. Primary analyses will use mixed-effects models to examine mental-health trajectories, with reproductive status modeled both as a baseline cohort characteristic and, where participants change status during follow-up, as a time-varying covariate; reproductive transitions will also be examined as secondary longitudinal outcomes.

**Discussion:**

This protocol addresses an important gap by using a longitudinal design to examine women’s experiences in a wartime setting. By situating mental health within disrupted reproductive plans, insecurity, healthcare strain, and family-level experiences, the study may help identify how armed conflicts shape both psychological vulnerability and reproductive decision-making. The findings may inform more responsive care for women affected by war.

**Clinical Trial Registration:**

ClinicalTrials.gov; identifier: NCT07551934.

## 1 Introduction

Although pregnancy and childbirth are often framed as celebratory and desirable life events, they are also periods of considerable burden associated with an elevated risk of mental health disorders. Meta-analytic evidence indicates that approximately 4.7% of mothers develop childbirth-related posttraumatic stress disorder (1), while clinically significant posttraumatic stress symptoms after childbirth have been reported in up to 16.8% of women (2). The prevalence of postpartum depression has been estimated at 14.0% to 24.7% (3–5), whereas anxiety disorders affect between 8.5% and 20.7% of pregnant or postpartum women (6–8). These disorders are further associated with pregnancy and obstetric complications (9–11), adverse developmental outcomes in children (12–14), and increased fear of subsequent childbirth (15, 16).

Such vulnerability is likely to be further exacerbated under conditions of armed conflict. Since the onset of Russia’s full-scale invasion of Ukraine on 24 February 2022, women in Ukraine have been experiencing pregnancy, childbirth, and motherhood in the context of repeated exposure to threat (17, 18), forced displacement (19, 20), family separation (21–23) and disruption of healthcare delivery (24–26). According to the United Nations Population Fund, 12.7 million people in Ukraine and 1.7 million Ukrainian refugees in Europe need reproductive and maternal health assistance (27). The World Health Organization has further documented at least 2,881 attacks on health care in Ukraine since the beginning of the full-scale invasion and reported that the number of such attacks in 2025 increased by nearly 20% compared with the previous year (28). Under such conditions, pregnancy and the postpartum period may unfold amid persistent anticipatory fear and uncertainty regarding maternal and infant safety (29).

These circumstances provide strong grounds to expect an intensification of psychological burden during the perinatal period, particularly when stress exposure is prolonged and embedded in everyday living conditions. In this protocol, such exposure is interpreted through the allostatic load framework, which conceptualizes chronic stress as a cumulative burden arising from repeated adjustments (30–32). In wartime, this burden may be sustained by recurrent air-raid alarms and explosions (33), displacement (34), separation from partners or relatives (35), financial insecurity (36), and direct or indirect exposure to injury or death (37). This is particularly relevant during pregnancy and postpartum, when maternal adaptation already requires coordinated changes (38). When these adaptive systems are repeatedly mobilized under conditions of prolonged threat, the resulting cumulative burden may contribute to allostatic overload and disrupt maternal–placental–fetal regulation, with potential consequences for perinatal mental health and obstetric outcomes (39, 40). Consequently, wartime perinatal distress should be considered within a cumulative-exposure framework.

This perspective draws attention to conditions that may either buffer or intensify the effects of chronic stress during pregnancy and the postpartum period. Relational and gendered safety is one such condition. Domestic and intimate partner violence are consistently associated with poorer perinatal mental health (41–43). Armed conflict may increase women’s vulnerability to such risks through economic dependence (44), partner trauma (45), and disruption of formal protection and support services (46). At the same time, the relevance of violence-related factors is not limited to direct victimization. Structural beliefs that minimize domestic violence, blame victims, or frame partner violence as a private family matter may reduce recognition, disclosure, and help-seeking (47). These factors are therefore relevant to perinatal mental health because they shape women’s perceived safety and ability to obtain timely protection or care in a wartime context.

Consistent with a cumulative-risk perspective, previous research has demonstrated that war and humanitarian crises adversely affect maternal and child health (48). However, the available evidence remains limited in several important respects. Most empirical studies on perinatal mental health have been conducted in high-income settings (49) characterized by comparatively stable healthcare systems (50) and greater continuity of obstetric care (51). By contrast, evidence from low- and middle-income countries and from populations directly exposed to ongoing armed conflict remains relatively sparse, notwithstanding indications of heightened vulnerability in such settings (5, 52, 53). Studies conducted in Israel have reported elevated risks of trauma-related outcomes (54), perinatal depression (55), adverse birth outcomes (56) or complications (57) in conflict-affected populations. Similarly, adverse effects of war on perinatal mental health have been documented among Syrian (58), Sudanese (59), Liberian (60), Afghan (61), and Pakistani (62) women.

In Ukraine, the relevance of this gap is underscored by rapid demographic deterioration and large-scale population displacement since the full-scale invasion. The United Nations Population Fund (UNFPA) reported that Ukraine’s population declined from 42 million in January 2022 to 35.8 million in July 2024, including 31.1 million people in government-controlled areas (63). These shifts are accompanied by large-scale displacement. Specifically, by December 2025, 5.86 million refugees from Ukraine were recorded globally, including approximately 5.3 million in Europe, while 3.7 million people remained internally displaced inside Ukraine (64). These demographic and displacement pressures also unfold within a deteriorating gendered safety context: in 2023, the National Police of Ukraine registered more than 291,000 reports of domestic violence, 20% more than in 2022, and the UN estimated that 2.4 million people in Ukraine, mostly women and girls, were experiencing or at risk of gender-based violence and required support services (65). The Ukrainian Government’s National Demographic Strategy through 2040 further recognizes intensified demographic challenges related to fertility intentions, migration, and demographic resilience (66). Despite stable marriage rates, many couples are postponing parenthood due to insecurity and limited access to prenatal care (67). Wartime survey data show widespread postponement of parenthood, 56.9% reported postponing childbirth until the end of the war, while a further 24.6% linked postponement to displacement (68). These micro-level shifts are mirrored in population-level trends. According to UNFPA, fertility in Ukraine has fallen to around one child per woman, and only about 176,700 children were born in 2024, roughly 35% fewer than in 2021 (69). If current trends persist, Ukraine is projected to face continued depopulation, and the birth rate may decline by nearly half by 2070 (67). The full-scale invasion has intensified an already severe demographic crisis. UNFPA has already estimated that Ukraine’s population has declined by approximately 10 million since 2014, with most of this decline occurring since February 2022 (70). Together, these patterns indicate that war-related disruption may affect reproductive decision-making, pregnancy planning, and family-planning trajectories, in addition to perinatal mental health. This is consistent with evidence from other conflict-affected settings, where disruptions to safety, predictability, and future orientation have been associated with the deferral of long-term life decisions, including childbearing (71, 72).

Research in humanitarian and conflict-affected settings remains methodologically limited, with much of the available evidence based on cross-sectional designs and comparatively few longitudinal studies (73, 74). Moreover, perinatal mental health and reproductive decision-making are typically examined separately, resulting in limited evidence on how psychological distress, fertility intentions, and pregnancy planning are associated with perinatal health outcomes (75). Against this background, a longitudinal study of reproductive behavior and perinatal mental health in wartime Ukraine is warranted. The present prospective cohort study aims to examine how war-related stressors interact with psychological, contextual, and sociodemographic factors in shaping reproductive intentions, reproductive transitions, and perinatal mental health among Ukrainian women. In doing so, the study seeks to address a substantial gap and to generate evidence capable of informing contextually grounded reproductive and perinatal care in war-affected settings.

On this basis, we hypothesize that higher war-related stress exposure will be associated with less favorable trajectories in the primary mental-health outcome family across follow-up. Second, we hypothesize that reproductive status and reproductive-status transitions will be associated with differences in mental-health trajectories and with secondary reproductive-behavior outcomes. Third, exploratory analyses will examine whether relational, sexual, and dyadic contextual measures, including domestic-violence myth acceptance, attitudes toward sex during pregnancy, motivations against sex, and partner-reported variables where available, are associated with mental-health trajectories and secondary reproductive-behavior outcomes.

## 2 Methods and analysis

### 2.1 Study design

The study is designed as a prospective observational cohort study with four waves of data collection: one baseline assessment and three follow-up assessments. The protocol was developed in accordance with the Standardized Protocol Items: Recommendations for Observational Studies (SPIROS) (76). The study will be conducted and reported in line with the STrengthening the Reporting of OBservational Studies in Epidemiology (STROBE; RRID: SCR_018788) guidelines (77). The study protocol was approved by the Bioethics Committee at Taras Shevchenko National University of Kyiv (No. 3/03-04-2026).

Participants will be enrolled on a rolling basis through healthcare institutions in Ukraine that provide reproductive and perinatal care. The study will be conducted in routine clinical settings without any modification to standard clinical management; accordingly, participation is expected to involve no more than minimal risk and a low procedural burden. Participants will not be assigned to treatment, and study participation will not modify routine clinical management. The study procedures are limited to informed consent, web-based questionnaire completion, optional follow-up contact, optional partner questionnaire completion, and, where specific consent is provided, extraction of predefined variables from routine clinical records. Any safety messages, crisis-resource information, or referral information in the questionnaire will be used as ethical risk-management procedures and will not constitute a study intervention. Participants may enter the study at different reproductive stages; however, once enrolled, all participants will be followed according to the same repeated-assessment framework. The baseline assessment will define cohort entry and serve as the reference point for reproductive status and the initial levels of the main study variables. Follow-up assessments are intended to capture change over time, including variation in outcome levels, changes in pregnancy intentions, transitions across reproductive stages, and temporal associations between contextual exposures and subsequent psychological outcomes.

Reproductive status will therefore be recorded at each assessment wave. Transitions in reproductive status during follow-up will be coded longitudinally. This approach will allow reproductive status to be incorporated both as a time-varying covariate in models of mental-health trajectories and, where appropriate, as a secondary time-varying outcome reflecting reproductive change during the study period. This rolling-entry longitudinal design supports both cross-sectional comparisons at baseline and prospective analyses of within-person change over time. As an observational cohort design, the study will allow prospective examination of temporal associations and within-person change, but it will not permit causal inference equivalent to an experimental design.

### 2.2 Study sample

The primary study sample will consist of women recruited across three reproductive-status groups: non-pregnant, pregnant, and postpartum women within 12 months after childbirth. These groups were defined to capture distinct stages across the reproductive and perinatal continuum. The inclusion of non-pregnant women ensures representation across the full range of reproductive stages (78) and increases the likelihood of observing changes in pregnancy-planning intentions during longitudinal data collection (79).

Where feasible, partners of participating women will also be recruited. Partner participation is intended to supplement the primary dataset by providing dyadic information on reproductive decision-making and mental health within the couple context. Accordingly, women constitute the primary target population of the study, whereas partners represent a secondary complementary subgroup linked to enrolled participants. Women will be eligible to participate and remain in the study regardless of whether their partner is invited, agrees to participate, completes the questionnaire, or remains in follow-up.

#### 2.2.1 Sample size

The sample size was estimated for the primary longitudinal mixed-model repeated-measures analysis, with the World Health Organization–Five Well-Being Index (WHO-5) score anchored as the primary outcome. Accordingly, the calculation was designed to provide adequate statistical power to detect differences in longitudinal WHO-5 trajectories according to war-related stress exposure. Sample size estimation was conducted in R (RRID: SCR_001905) using the *longpower* package (80). Parameter values were derived from previously published longitudinal studies, consistent with Harrall et al.’s recommendations (81). Given the substantial logistical and participant burden associated with data collection during an ongoing war, statistical power was set at 0.8, and the type I error rate was set at 0.05. Equal allocation across the two comparison levels specified in the power model was assumed.

The standard deviation of the WHO-5 outcome among postpartum women was set at 1.05, based on data reported by Mor et al. (82). The correlation structure was specified as a first-order autoregressive process with a correlation parameter of 0.38, informed by longitudinal data reported by Qandil et al. (83). Retention assumptions were based primarily on the cumulative follow-up pattern reported by Damtew et al. (84), with retention rates of 92.9% at 6 weeks, 84.2% at 6 months, and 73.0% at 12 months, calculated using the baseline cohort as the denominator at each follow-up. Although some studies in post-conflict settings have reported relatively high retention, such as the 77.3% 2-year retention reported by Silove et al. (85), the present assumptions were considered more appropriate for this study context.

The final sample size calculation assumed a heterogeneous first-order autoregressive variance-covariance structure, four measurement occasions, and a two-sided test. Under these assumptions, the minimum required sample size was estimated at 328 participants. This sample size is expected to provide adequate power for detecting differences in longitudinal WHO-5 trajectories by war-related stressor exposure in the primary mixed-model repeated-measures analysis after accounting for attrition. The sample-size calculation was anchored to the WHO-5 score as the reference outcome for the primary longitudinal analysis in the women’s cohort. Secondary outcomes, for instance, reproductive-behavior outcomes, clinical outcomes, reproductive-status-by-time interactions, and the optional dyadic partner component will be analyzed where the available data structure, sample size, and model stability permit. These analyses will not be considered independently powered confirmatory tests; they will be interpreted as hypothesis-generating.

A sensitivity power analysis was also conducted using a more conservative compounded attrition scenario, assuming 85% retention per 3-month intervals up to the 12-month follow-up, to reflect the possibility of greater attrition in the wartime context. In this scenario, retention proportions were set at 1.00, 0.85, 0.72, and 0.52. Under this conservative attrition scenario, the planned sample size of N = 328 would provide approximately 70% power, which was considered relevant for a perinatal population in a wartime setting (86). To maintain 80% power under the same conservative retention assumptions, the required sample size would increase to approximately N = 416. Accordingly, the planned sample size should be interpreted as adequately powered for the primary longitudinal model under the main retention assumptions, whereas findings should be interpreted more cautiously if attrition approaches the conservative scenario. The corresponding R script is available in the project Open Science Framework (RRID: SCR_003238) repository (87).

#### 2.2.2 Eligibility criteria

Eligible participants will include women receiving gynecological or perinatal healthcare services at participating recruitment sites. Women will be enrolled into one of three baseline reproductive-status cohorts: non-pregnant women, pregnant women, or postpartum women within 12 months after childbirth. For the purposes of this study, the non-pregnant cohort will include women who are not currently pregnant and are not within 12 months postpartum, but who are attending reproductive or gynecological healthcare and meet at least one of the following criteria: actively planning pregnancy, receiving preconception counseling, receiving fertility or infertility assessment, seeking contraception counseling, prescription, continuation, removal, or change, receiving care related to previous pregnancy loss, or attending reproductive-health care where contraception or pregnancy avoidance is clinically relevant. Current intention to conceive will not be required for inclusion in this group. Pregnant women will be eligible at any gestational stage. Postpartum women will be eligible if they are within 12 months after childbirth at the time of baseline recruitment. For all postpartum participants, the date of childbirth or infant age in months will be recorded to determine time since childbirth.

Pregnancy intention, current contraception status, fertility or infertility treatment, pregnancy-loss history, and reproductive-status category will be recorded at baseline and updated during follow-up where applicable. These characteristics will be used to describe the cohort and to support planned adjustment, subgroup, or sensitivity analyses where data structure and model stability permit; they will not serve as independent exclusion criteria unless participation falls outside the study’s reproductive or perinatal healthcare focus. Where feasible, partners of enrolled women will also be recruited as a supplementary subgroup. All participants will be eligible for inclusion if they meet all the following criteria: able and willing to provide informed consent, and aged 18 years or older. Individuals outside the reproductive or perinatal care pathway relevant to the study will not be eligible.

Participants will be excluded if any of the following apply: they are younger than 18 years; they are unable to provide informed consent; they do not have sufficient language proficiency to complete the study procedures; at the time of recruitment, they have an acute medical or psychological condition that would make participation unsafe, inappropriate, or unduly burdensome (e.g., obstetric emergencies, immediate postoperative or critical care status, or severe acute distress requiring urgent clinical care); or participation or the handling of personal data could reasonably pose a risk to the safety of the participant or their family. If the acute condition resolves and the individual remains otherwise eligible, they may be invited to consider participation at a later recruitment opportunity.

Participants who experience pregnancy loss, stillbirth, neonatal loss, severe obstetric complications, or traumatic birth events during follow-up will not be excluded from the study on this basis. Where such events occur, reproductive and perinatal status will be updated at the next assessment wave, and questionnaire routing will be adapted to avoid non-applicable items. Participants may continue follow-up, skip any question, pause participation, or withdraw without consequence. If participation is considered unsafe or unduly burdensome because of acute distress at the time of assessment, follow-up may be temporarily deferred, and the participant may be re-contacted later where appropriate and safe.

These criteria were defined *a priori* during protocol development based on the study objectives, the intended clinical context, the requirements of the selected instruments, and ethical principles for research involving human participants. The eligibility framework is intended to ensure that participants are both appropriate to the study aims and able to provide valid self-report data, while minimizing burden and protecting individuals in situations of acute medical, psychological, or contextual vulnerability. Eligibility will be assessed during initial screening by trained study personnel, and final enrollment will occur only after the prospective participant has received the participant information sheet, had an opportunity to ask questions, and provided written or electronically documented informed consent in accordance with approved study procedures.

#### 2.2.3 Recruitment procedure

Potential participants will be identified during attendance for relevant reproductive or perinatal healthcare services. Recruitment will follow a consecutive invitation strategy, whereby all individuals meeting eligibility criteria during predefined recruitment days and/or shifts will be systematically invited to participate. The initial approach will be made by a healthcare professional not directly involved in the participant’s treatment or assessment. This procedure is intended to minimize selection bias and reduce the possibility of perceived pressure to participate.

At the time of invitation, potential participants will be informed about the purpose of the study, the nature of the questionnaire, the estimated duration of participation, the voluntary nature of participation, and the absence of any consequences for medical care in the event of refusal or withdrawal. Participants who agree to take part will be provided with the participant information sheet and asked to complete informed consent before accessing the questionnaire.

Partner recruitment will occur only after the woman has been informed that partner participation is optional and has had an opportunity to consider this separately from her own enrollment. Women will be able to decline partner involvement without giving a reason, and this decision will not be disclosed to partners, accompanying persons, or clinical staff involved in their care. Where partner recruitment is considered appropriate, partners will receive separate study information, provide independent informed consent, and complete the questionnaire separately. Partner invitation will not be pursued if privacy cannot be ensured or if there is any indication that partner involvement could increase risk, pressure, or discomfort for the woman, including concerns related to intimate partner violence, reproductive coercion, family separation, or conflict-related stress.

**Figure 1:**
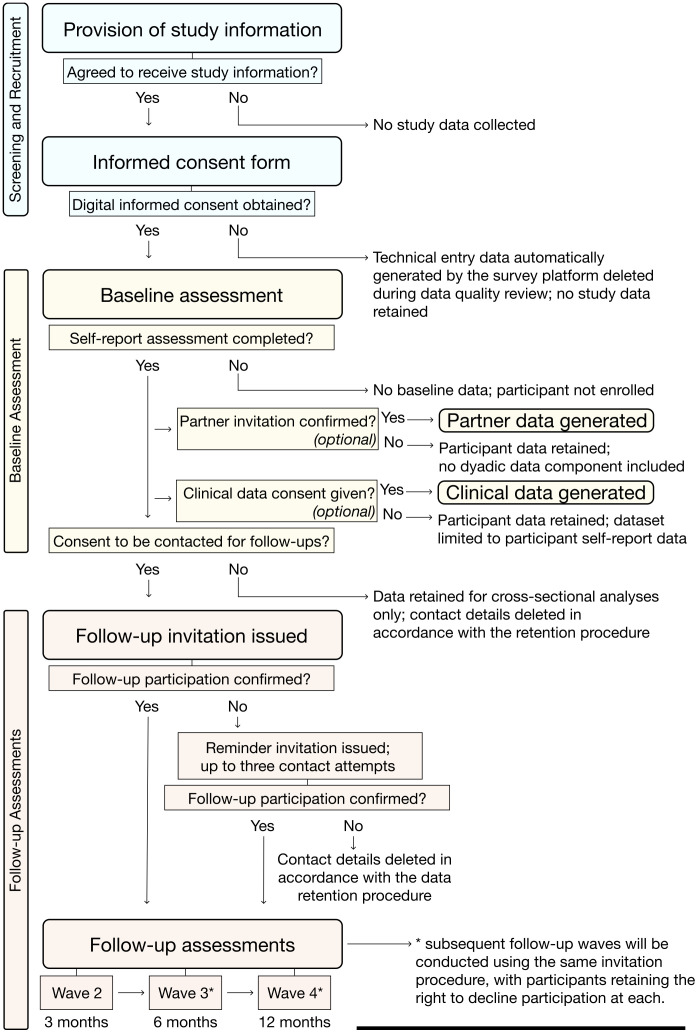
Participant’s journey flowchart.

To promote consistent recruitment, no targeted selection will be undertaken based on psychological condition, trauma history, or other non-eligibility characteristics; instead, all potentially eligible individuals will be approached according to the same predefined procedure. The participant’s journey is summarized in **Figure 1**.

### 2.3 Data collection

Data will be collected using web-based questionnaires administered via the 1KA (RRID: SCR_019283) survey platform. Participants will access the survey through a QR code or direct hyperlink and may complete it either on site at the recruiting healthcare facility or remotely. The study will use two digital data collection instruments. The first will be a participant-completed questionnaire administered to eligible women and, where applicable, to partners included in the supplementary dyadic component. Question flow will be adapted through programmed routing logic so that participants are presented only with items relevant to their reproductive stage and experiences. The second instrument will be completed by authorized healthcare personnel only where participants have provided specific consent for such data collection. This instrument will be used to record relevant clinical information derived from routine care, including findings from medical observation, functional assessment, and laboratory or instrumental examinations, where available within the clinical setting. No biological specimens will be collected, processed, or stored specifically for the purposes of this study.

Following completion of the baseline survey, participants will be invited to provide contact details for the purpose of re-contact and follow-up participation. Follow-up invitations will be scheduled relative to each participant’s baseline completion date. The target assessment points will be 3, 6, and 12 months after baseline, with acceptable completion windows defined as 2.5–3.5 months for the 3-month assessment, 5–7 months for the 6-month assessment, and 10–14 months for the 12-month assessment. The exact date of questionnaire completion and the number of days since baseline will be recorded for each wave. Assessments completed outside these windows will be flagged during data management and considered in sensitivity analyses where relevant. In longitudinal models, time will be represented primarily by assessment wave, with elapsed time since baseline examined where deviations from the target schedule are substantial. Participants who agree to follow-up contact will receive invitations by email at each follow-up wave. Contact details collected for this purpose will be stored separately from questionnaire responses and will not be incorporated into the analytical dataset. Participants will retain the right to decline participation at any follow-up wave without consequence. The total duration of data collection is expected to range from approximately 3 to 12 months, depending on recruitment rates and follow-up completion.

To support study administration and data quality monitoring, the survey platform will record limited process metadata, including date of survey completion, response status, and item-level missingness. These metadata will be used solely for monitoring completeness, identifying duplicate or invalid entries where applicable, and supporting data quality review. At the end of the questionnaire, participants will be provided with information on relevant mental health and lactation support resources, together with contact details for the research team.

To enable confidential linkage of dyadic data while minimizing identifiability, participant records within couples will be matched using a self-generated identification code (SGIC). Each member of the couple will complete the survey independently. The identification code will be constructed according to the procedure described by Harani and Ben-Porat (88), whereby each couple generates a ten-character code composed of the first initials of both partners and their respective two-digit birth months and days (e.g., NS04231017). The first respondent will share the code with the partner to facilitate accurate matching, and a designated questionnaire item will distinguish respondents within the dyad. Because generating or sharing a dyadic code may create privacy or safety concerns in some relationships, participants will be instructed to proceed with partner linkage only if this can be done safely and voluntarily. If sharing the code would be uncomfortable, unsafe, or impractical because of conflict-related separation, relationship strain, domestic violence, reproductive coercion, or lack of privacy, the participant may complete the questionnaire without partner linkage. Cases that cannot be matched at the dyadic level will be retained for individual-level analyses where otherwise eligible but will be excluded from analyses requiring paired data. This approach safeguards participant anonymity while facilitating dyadic analysis and aligns with the Sex and Gender Equity in Research (SAGER) guidelines (89).

All data will be collected in anonymized form, with only indirect identifiers retained for dyadic matching. Any contact information collected for follow-up purposes will be stored separately from survey responses and will be accessible only to authorized members of the research team. Variables used for dyadic linkage or follow-up administration will be retained only for as long as necessary to complete matching and longitudinal follow-up procedures and will subsequently be deleted from the working dataset and stored separately under restricted access, in accordance with the study Data Management Plan (87).

### 2.4 Measures

The study will employ a combination of self-report questionnaires and clinician-recorded indicators to assess the variables of interest. The characteristics of the selected measures are presented in **Table 1**, whereas the available evidence regarding the validity and reliability of the measures is summarized in **Table 2**. The assessment schedule, including variations by cohort and wave of data collection, is presented in **Table 3**. Where no validated Ukrainian-language version of a measure was available, translation was undertaken during protocol development by members of the study team, Liudmyla Krupelnytska, Olha Morozova-Larina, and Nazar Yatsenko, in accordance with Brislin’s framework (90) and Cruchinho et al.’s recommendations (91). Before the launch of the main cohort study, translated and adapted measures will undergo a preliminary pilot-testing phase to assess item clarity, cultural and contextual appropriateness, acceptability, survey routing, completion burden, and technical functioning of the web-based questionnaire. Feedback from this phase will be used to make minor wording or procedural refinements before the instruments are used in the longitudinal cohort. This pilot phase is intended to support feasibility and content clarity rather than to replace formal psychometric validation. The psychometric evaluation of these Ukrainian-language versions is planned as part of the main study, where adequate sample size is available, and will be reported separately.

**Table 1:** Characteristics of the included measures.

Measure	Source		Items	Response format	Number of Factors	Factor structure	Score range	Cut-off value
	Original measure	Ukrainian version						
ACT-PNM	Rodríguez-Muñoz et al. (2023) (86)	Rodríguez-Muñoz et al. (2023) (86)	40	Mixed	N/A	N/A	N/A	N/A
WHO-5	World Health Organization (1998) (100)	Lotzin et al. (2023) (73)	5	Likert scale (0–5)	1	Unidimensional	0–25 (0–100)*	50*
EPDS	Cox et al. (1987) (104)	Chrzan-Dętkoś et al. (2026) (111)	10	Likert scale (0–3)	3	Anhedonia; anxiety; depression	0–30	13
City BiTS	Ayers et al. (2018) (108)	Chrzan-Dętkoś et al. (2026) (111)	29	Likert scale (0–3)	2/4	2-factor: Birth-related symptoms; general symptoms. 4-factor: Re-experiencing; avoidance; negative cognitions and mood; hyperarousal	0–60	29
City BiTS (Partner Version)	Webb et al. (2021) (126)	—	29	Likert scale (0–3)	2/4	2-factor: Birth-related symptoms; general symptoms. 4-factor: Re-experiencing; avoidance; negative cognitions and mood; hyperarousal	0–60	—
BSS-R	Martin & Martin (2014) (130)	—	10	Likert scale (0–4)	3	Quality of care provision; women’s personal attributes; stress experienced during labor	0–40	—
DAP	Rocca et al. (2019) (118)	—	14	Likert scale (0–4)	3	Cognitive desires and preferences; affective feelings and attitudes; anticipated practical consequences	0–4	—
LMUP	Barrett et al. (2004) (121)	—	6	0–2	1	Unidimensional	0–12	—
MSP/PSP	Jawed-Wessel et al. (2016) (135)	—	MSP: 6 PSP: 8	Likert scale (1–6)	1	Unidimensional	1–6	—
MASQ	Patrick et al. (2011) (138)	—	9	Likert scale (1–5)	3	Values; health; not ready	1–5	—
DVMAS	Peters (2008) (139)	—	18	Likert scale (1–7)	4	Minimization; exoneration; behavior blame; character blame	1–7	—
GAD-7	Spitzer et al. (2006) (112)	Aleksina et al. (2024) (115)	7	Likert scale (0–3)	1	Unidimensional	0–21	7
IES-R	Weiss & Marmar (1997) (92)	Krupelnytska et al. (2025) (97)	22	Likert scale (1–5)	3	intrusion; avoidance; hyperarousal	22–110	30
SCI-2-SF	De Luca et al. (2024) (116)	Yatsenko et al. (2024) (117)	9	Likert scale (0–4)	5	Entrapment; affective disturbance; loss of cognitive control; hyperarousal; social withdrawal	0–4	—
ACE	Felitti et al. (1998)	Lotzin et al. (2023) (73)	10	Dichotomous	N/A	N/A	0–10	N/A

*, raw WHO-5 multiplied by 4 to obtain standardized score ranging from 0 to 100; the cut-off value of 50 refers to the transformed 0–100 score.

**Table 2:** Psychometric evidence for the study measures.

Measure	Version	Validation source	Internal consistency	Test–retest/procedural reliability	Item-level evidence	Factor structure/model fit	Validity/diagnostic accuracy
WHO-5	Original	World Health Organization (1998) (100)	α = .84	Not reported	Not reported	Loevinger’s H = .56	Not reported
EPDS	Original	Cox et al. (1987) (104)	α = .87; split-half reliability = .88	Not reported	Not reported	Not reported	Sensitivity = .86; specificity = .78; PPV = .73
	Ukrainian	Chrzan-Dętkoś et al. (2026) (111)	α = .86–.87; subscale α = .73–.85	Not reported	Item-total r = .35–.73	CFA: χ²(32) = 55.34–79.29, CFI = .97, TLI = .96, RMSEA = .06, SRMR = .04	Convergent validity: r = .44–.72
City BiTS	Original	Ayers et al. (2018) (108)	α = .92; subscale α = .83–.88	Not reported	Not reported	KMO = .94; Bartlett’s χ²(190) = 10,283.1, p <.001; EFA supported a two-factor structure, explaining 56.3% of variance	Convergent validity: r = .50–.61
	Ukrainian	Chrzan-Dętkoś et al. (2026) (111)	α = .92; ω = .92; subscale α = .73–.91; subscale ω = .80–.92	Not reported	Item-total r = .27–.76	CFA: CFI = .949, TLI = .932, RMSEA = .062, AIC = 450.29; ECV = .544, ωH = .635, H = .916	Convergent validity: r = .36–.53; divergent validity: r = .31–.61
City BiTS (Partner version)	Original	Webb et al. (2021) (126)	α = .94; subscale α = .78–.92	Not reported	Not reported	KMO = .926; Bartlett’s χ²(190) = 3745.14, p <.001; EFA supported a two-factor structure, explaining 50.81% of variance	Not reported
BSS-R	Original	Martin & Martin (2014) (130)	α = .79; subscale α = .64–.74	Not reported	Not reported	CFA: χ²(32) = 42.67, p = .10, χ²/df = 1.33, CFI = .98, RMSEA = .04, RMR = .04, SRMR = .05	Not reported
DAP	Original	Rocca et al. (2019) (118)	α = .95–.96; separation reliability = .90–.91	ICC = .95	Item-total r = .53–.83	Not reported	Sensitivity = .78; specificity = .81
LMUP	Original	Barrett et al. (2004) (121)	α = .92	Test–retest κ = .97; long-term κ = .86	Item-total r = .60–.89	PCA supported a one-factor structure; eigenvalue = 4.33; loadings = .70–.93	Not reported
MSP/PSP	Original	Jawed-Wessel et al. (2016) (135)	MSP: α = .89; PSP: α = .91	Not reported	Factor loadings: MSP = .74–.85; PSP = .77–.92	EFA supported a one-factor structure for MSP, explaining 64.58% of variance, and a one-factor structure for PSP, explaining 69.35% of variance	Not reported
MASQ	Original	Patrick et al. (2011) (138)	α = .75–.94	Not reported	Not reported	EFA supported a six-factor structure, explaining 66.83% of variance; CFA: CFI = .93–.94, RMSEA = .03–.05	Not reported
DVMAS	Original	Peters (2008) (139)	α = .88	Not reported	Not reported	Four-factor structure reported	Correlations with related constructs: RMA r = .65, SRS r = .51, ATW r = .47, AWA r = .37
GAD-7	Original	Spitzer et al. (2006) (112)	α = .92	Retest ICC = .83; procedural ICC = .83	Not reported	Not reported	Sensitivity = .89; specificity = .82
	Ukrainian	Aleksina et al. (2024) (115)	α = .88–.90; ω = .87–.89; λ6 = .88–.90; split-half reliability = .83–.87	Retest r = .72	Item-total r = .73–.85	EFA supported a one-factor structure, explaining 52.3%–57.1% of variance; CFA: CFI = .973–.985, TLI = .959–.977, RMSEA = .064–.078	Not reported
IES-R	Original	Weiss & Marmar (1997) (92)	α = .79–.92	Retest r = .51–.94	Not reported	EFA supported a three-factor structure	Not reported
	Ukrainian	Krupelnytska et al. (2025) (97)	α = .91; ω = .92; subscale α/ω = .75–.84	Retest ICC = .206; retest r = .412	Not reported	CFA: CMIN/DF = 2.874, RMSEA = .049, SRMR = .065, CFI = .977, TLI = .974	Not reported
SCI-2-SF	Original	De Luca et al. (2024) (116)	Not reported	Not reported	Not reported	Not reported	AUROC = .824–.856
	Ukrainian	Yatsenko et al. (2024) (117)	α = .88; ωH = .84; subscale α = .43–.79	Not reported	Factor loadings >.40	CFA: RMSEA = .033, CFI = .98, TLI = .98, SRMR = .05; MSA = .91	Convergent validity: r = .210–.421; divergent validity: r = −.358

α, Cronbach’s alpha; ω, McDonald’s omega; ωH, hierarchical omega; λ6, Guttman’s lambda 6; ICC, intraclass correlation coefficient; κ, kappa coefficient; PPV, positive predictive value; EFA, exploratory factor analysis; CFA, confirmatory factor analysis; PCA, principal component analysis; KMO, Kaiser–Meyer–Olkin measure; CFI, comparative fit index; TLI, Tucker–Lewis index; RMSEA, root mean square error of approximation; SRMR, standardized root mean square residual; RMR, root mean square residual; AIC, Akaike information criterion; ECV, explained common variance; H, construct replicability index; MSA, measure of sampling adequacy; AUROC, area under the receiver operating characteristic curve; RMA, rape myth acceptance; SRS, sex-role stereotyping; ATW, attitudes toward women; AWA, adversarial sexual beliefs.

**Table 3:** Schedule of measure administration across study assessment waves.

Measure	Cohort															
	Non-pregnant women				Pregnant women				Postpartum women				Partners			
	T1	T2	T3	T4	T1	T2	T3	T4	T1	T2	T3	T4	T1	T2	T3	T4
Participant-completed measures																
ACT-PNM	+	+	+	+	+	+	+	+	+	+	+	+	+	+	+	+
WHO-5	+	+	+	+	+	+	+	+	+	+	+	+	+	+	+	+
EPDS					+	+	+	+	+	+	+	+				
City BiTS									+	+	+	+				
City BiTS (Partner version)													+	+	+	+
BSS-R									+	+	+	+				
DAP	+	+	+	+												
LMUP					+	+		+	+	+		+				
MSP/PSP					+	+	+	+					+	+	+	+
MASQ	+			+	+	+	+	+	+	+	+	+	+	+	+	+
DVMAS	+			+	+			+	+			+	+			+
GAD-7	+	+	+	+	+	+	+	+	+	+	+	+	+	+	+	+
IES-R	+	+	+	+	+	+	+	+	+	+	+	+	+	+	+	+
SCI-2-SF	+	+	+	+	+	+	+	+	+	+	+	+	+	+	+	+
ACE	+				+				+				+			
Clinician-recorded measures																
Laboratory assessment	+	+	+	+	+	+	+	+	+	+	+	+				
Imaging assessment	+				+				+							
Obstetric parameters					+	+	+	+								
Fetal assessment					+	+	+	+								
Perinatal parameters									+							
Neonatal screening									+							

#### 2.4.1 Exposure measures

##### 2.4.1.1 Assessment, care, and trust – in pregnant and new mothers

At baseline, all participants will be assessed using the Assessment, Care, and Trust in Pregnant and New Mothers (ACT-PNM) questionnaire developed by Rodríguez-Muñoz et al. (86). This 40-item instrument collects demographic and background information and assesses war-related experiences. To ensure applicability across all study cohorts, the questionnaire was slightly modified for use with women’s partners and non-pregnant women. Data derived from this measure will be used to describe the study sample and examined as potential covariates in further analyses. Where relevant, selected war-related items will be repeated at follow-up assessments to capture changes in participants’ living circumstances over time in the context of ongoing war. A psychometric evaluation of the questionnaire is not required, because the instrument was designed to capture a broad range of living and reproductive experiences in wartime Ukraine and not to measure a single latent construct or generate a unified scale score (86).

War-related stressor exposure will be assessed using predefined ACT-PNM indicators reflecting discrete wartime experiences and living conditions. For the primary analysis, war-related stressor burden will be operationalized as the number of endorsed stressor domains at baseline, with higher values indicating exposure to a broader range of war-related stressors. The predefined domains will include displacement, separation from a partner or close family members, disruption of healthcare access, economic hardship, proximity to hostilities or unsafe living conditions, and direct or indirect exposure to threat, injury, death, or war-related attacks. Domain-specific indicators will also be retained for secondary analyses to examine whether forms of exposure are differentially associated with mental-health trajectories.

Because exposure may change during ongoing war, selected ACT-PNM indicators will be reassessed at follow-up waves where applicable. In secondary longitudinal analyses, time-updated war-related stressor burden will be modeled as a time-varying exposure, defined as the cumulative number of stressor domains reported up to each assessment point. These analyses will be interpreted as secondary because war-related circumstances and psychological symptoms may change concurrently during follow-up. The IES-R will not be used to define war-related exposure, as it measures trauma-related symptom severity rather than exposure itself.

##### 2.4.1.2 Impact of events scale – revised

The Impact of Event Scale – Revised (IES-R) is a self-report questionnaire used to assess trauma-related symptoms following exposure to a traumatic event (92). The instrument comprises 22 items, rated on a 5-point Likert scale from 1 to 5, with higher scores indicating greater trauma-related symptom severity and a cut-off score of 30 indicating a high probability of a trauma-related disorder. The scale has been widely used in perinatal populations to assess distress in the context of the COVID-19 pandemic (93–95) as well as disasters (96). The validated Ukrainian version of the measure will be used in the present study (97).

##### 2.4.1.3 Adverse childhood experiences questionnaire

The Adverse Childhood Experiences Questionnaire (ACE) is a self-report questionnaire used to assess exposure to adverse experiences before the age of 18 years. Items are answered dichotomously, yielding a cumulative score, with higher scores indicating exposure to a greater number of adverse childhood experiences. The adverse childhood experiences framework has demonstrated a graded association with multiple adult health-risk behaviors and health outcomes (98). In the present study, the Ukrainian version of the measure will be used to assess retrospective exposure to childhood adversity. The Ukrainian version used in the present study was provided for Lotzin et al.’s study (99), although no prior validation studies have been published.

#### 2.4.2 Primary outcome measures

##### 2.4.2.1 World health organization – five well-being index

The WHO-5 is a brief self-report measure developed to assess psychological well-being (100, 101). The instrument comprises five items rated on a 6-point Likert scale from 0 to 5, yielding a raw total score ranging from 0 to 25, with higher scores indicating greater well-being. In accordance with standard scoring procedures, the raw total score is multiplied by 4 to produce a final score, with a score of 50 or below used as the cut-off for significantly reduced well-being, in line with the recommendation of Topp et al. (102). This instrument has also been identified as a promising measure for use in pregnant women (100), particularly due to its potential utility in predicting depressive symptoms (103). The Ukrainian translation of the measure used in the present study was the version provided for Lotzin et al.’s study (99), for which no prior validation studies have been published.

##### 2.4.2.2 Edinburgh postnatal depression scale

The Edinburgh Postnatal Depression Scale (EPDS; RRID: SCR_003685) is a self-report questionnaire developed to assess depressive symptoms during the perinatal period (104). The instrument comprises 10 items rated on a 4-point Likert scale from 0 to 3, yielding a total score ranging from 0 to 30, with higher scores indicating greater severity of depressive symptoms. A score of 13 or above will be used as the cut-off for clinically significant depressive symptoms, in line with the original recommendation of Cox et al. (104) and supported by Levis et al. (105). The scale has demonstrated good psychometric properties, including good internal consistency, sensitivity, and specificity in the original validation study, and has since been widely used in both antenatal and postnatal populations. The Ukrainian version of the EPDS used in the present study was developed and validated by Chrzan-Dętkoś et al. (106), and, in line with Lautarescu et al. (107), the three-factor model will be applied in the present study.

##### 2.4.2.3 City birth trauma scale

The City Birth Trauma Scale (City BiTS) is a self-report questionnaire developed to assess childbirth-related posttraumatic stress disorder symptoms in accordance with DSM-5 criteria (108, 109). The instrument comprises 29 items, with 20 symptom items rated on a 4-point Likert scale from 0 to 3. The symptom score ranges from 0 to 60, with higher scores indicating greater severity of childbirth-related posttraumatic stress symptoms. A score of 29 or above will be used as the cut-off for clinically significant childbirth-related posttraumatic stress symptoms, in accordance with the recommendation by Osório et al. (110). The Ukrainian version of the City Birth Trauma Scale used in the present study was developed and validated by Chrzan-Dętkoś et al. (111). In the present study, both factor-scoring approaches will be applied: the two-factor structure differentiating birth-related and general symptoms, and the four-factor structure differentiating DSM-5 symptom clusters.

##### 2.4.2.4 General anxiety disorder – 7

The Generalized Anxiety Disorder – 7 (GAD-7; RRID: SCR_003666) is a self-report questionnaire developed to assess symptoms of generalized anxiety disorder and general anxiety severity (112). The instrument comprises 7 items rated on a 4-point Likert scale from 0 to 3, yielding a total score ranging from 0 to 21, with higher scores indicating greater anxiety symptom severity. In the original validation study, a cut-off score of ≥10 was recommended. In the present study, a cut-off score of ≥7 will be applied for the perinatal population according to Gong et al. (113) and Zhong et al. (114). The Ukrainian adaptation provided by Aleksina et al. (115) demonstrated good internal consistency, test-retest reliability, and support for a one-factor structure.

##### 2.4.2.5 Suicide crisis inventory-2 short form

The Suicide Crisis Inventory – 2 Short Form (SCI-2-SF) is a self-report questionnaire developed to assess symptoms of the suicide crisis syndrome (116). The instrument assesses five symptom domains: entrapment, affective disturbance, loss of cognitive control, hyperarousal, and social withdrawal. The measure comprises 9 items rated on a 5-point Likert scale from 0 to 4, with higher scores indicating greater severity of suicidal crisis symptoms. The Ukrainian version of the measure was adapted and validated and demonstrated acceptable psychometric properties (117).

#### 2.4.3 Secondary outcome measures: reproductive behavior

##### 2.4.3.1 Desire to avoid pregnancy scale

The Desire to Avoid Pregnancy Scale (DAP) is a self-report measure developed to prospectively assess pregnancy preferences among individuals who could become pregnant (118). The instrument comprises 14 items rated on a 5-point Likert scale from 0 to 4. Item scores are averaged to produce a total score ranging from 0 to 4, with higher scores indicating a stronger desire to avoid pregnancy. The scale has demonstrated strong psychometric properties in the original validation study (118) and subsequent UK evaluation (119). A cut-point of <2 on the scale has been reported for predicting pregnancy within 12 months, with sensitivity of.78 and specificity of.81 (120). In the present study, the Ukrainian translation of the scale was prepared by the authors, and its psychometric properties will be evaluated within the current sample.

##### 2.4.3.2 London measure of unplanned pregnancy

The London Measure of Unplanned Pregnancy (LMUP) is a self-report instrument developed to assess the degree to which a pregnancy was planned (121). The instrument comprises six items, each scored from 0 to 2, yielding a total score ranging from 0 to 12, with higher scores indicating greater pregnancy planning. The original validation study demonstrated strong psychometric properties, including high internal consistency, test–retest reliability, and construct validity (121). The measure has also been validated in several languages and contexts, including Italian (122), Krio (123), and Turkish (124), and has been shown to be informative when integrated into routine antenatal care (125). In the present study, the Ukrainian translation of the measure was prepared by the authors, and its psychometric properties will be evaluated within the current sample.

#### 2.4.4 Exploratory measures

##### 2.4.4.1 City birth trauma scale (partner version)

The City Birth Trauma Scale Partner version (City BiTS (Partner version)) is a self-report questionnaire adapted from the original City Birth Trauma Scale to assess childbirth-related posttraumatic stress disorder symptoms in fathers and birth partners (126). Like the original City Birth Trauma Scale, the partner version is structured according to DSM-5 criteria and includes items assessing posttraumatic stress disorder symptoms, symptom duration, distress and functional impairment, and exclusion criteria. Evidence from the French adaptation indicates adequate psychometric performance (127), while Portuguese (128) and Norwegian (129) versions are currently in development. For the present study, a Ukrainian translation was prepared by the authors, and its psychometric properties will be evaluated as part of the current study.

##### 2.4.4.2 Birth satisfaction scale-revised

The Birth Satisfaction Scale-Revised (BSS-R) is a self-report questionnaire developed to assess women’s satisfaction with childbirth (130). The instrument comprises 10 items rated on a 5-point Likert scale and scored from 0 to 4, yielding a total score ranging from 0 to 40, with higher scores indicating greater birth satisfaction. The scale includes three subscales: quality of care provision, women’s personal attributes, and stress experienced during labor, which will be accounted for in the study alongside total score according to Martin et al.’s recommendation (131). The original validation study supported a three-factor structure and acceptable psychometric properties (130), with subsequent evidence from Croatian (132), Australian (133), and Turkish (134) adaptations further supporting the scale’s psychometric performance. The Ukrainian translation used in the present study was prepared by the authors, and its psychometric properties will be evaluated within the current study.

##### 2.4.4.3 Maternal and partner sex during pregnancy scales

The Maternal Sex During Pregnancy Scale (MSP) and Partner Sex During Pregnancy Scale (PSP) are self-report instruments developed to assess attitudes toward sex during pregnancy among pregnant women and their partners (135). The scale for mothers includes 6 items, and the one for fathers includes 8 items assessing aspects of pregnancy-related sexual attitudes. Items are rated on a 6-point Likert scale from 1 to 6, and item scores are averaged to produce a global attitude score ranging from 1 to 6. Higher scores indicate more positive attitudes toward sex during pregnancy. In the original validation study, both scales demonstrated a unidimensional structure and high internal consistency (135). Subsequent studies have supported the use of these measures among a Portuguese-speaking population (136) and Muslim women (137). For the present study, Ukrainian translations of the measures were prepared, and their psychometric properties will be evaluated within the current sample.

##### 2.4.4.4 Motivations against sex questionnaire

The Motivations Against Sex Questionnaire (MASQ) is a self-report measure developed to assess motivations for not engaging in sexual behavior. The instrument was developed as a complement to measures of motivations for sexual behavior and captures reasons for sexual avoidance (138). The MASQ comprises 9 items, with three items in each factor, rated on a 5-point Likert scale from 1 to 5. Subscale scores are calculated as the mean of the corresponding items, with higher scores indicating stronger endorsement of the respective motivation against sexual behavior. In the present study, the Ukrainian translation of the MASQ was prepared by the authors, and its psychometric properties will be evaluated within the current sample.

##### 2.4.4.5 Domestic violence myth acceptance scale

The Domestic Violence Myth Acceptance Scale (DVMAS) is a self-report instrument developed to assess endorsement of myths about domestic violence (139). The scale comprises 18 items. The original validation supported a multidimensional structure comprising character blame, behavior blame, minimization, and exoneration of the perpetrator. Higher scores indicate stronger endorsement of domestic violence myths. Evidence from French (140) and Portuguese (141) adaptations has further supported the reliability of the measure. For the present study, a Ukrainian translation was prepared by the authors, and its psychometric properties will be evaluated within the current sample.

#### 2.4.5 Clinical measures

Clinical data will be extracted from routine medical records only where participants provide specific consent and where the relevant information is available within the participating healthcare facility. The clinical-record component is intended to contextualize self-report data and to provide secondary information. Clinical-record variables will not constitute the primary outcome of the study.

Clinical variables will be divided into a core clinical dataset and an extended clinical dataset. The core clinical dataset will include variables required to characterize reproductive and perinatal status and to support adjustment or subgroup description, including current pregnancy status, gestational age where applicable, parity, time since childbirth for postpartum participants, pregnancy complications where recorded, delivery date and mode of delivery where applicable, and basic neonatal indicators such as gestational age at birth, birth weight, and Apgar scores where available. These variables will be prioritized across sites because they are most directly relevant to reproductive-status classification, cohort description, and interpretation of perinatal outcomes.

The extended clinical dataset will include additional variables available from routine care, grouped into the following domains: (a) maternal laboratory and biological indicators, including molecular and immunological markers, thyroid ultrasound findings, hormones, vitamins, and routine blood and urine test results; (b) pregnancy and obstetric characteristics, including maternal hemodynamic parameters, and pregnancy complications; (c) fetal antenatal assessment, including fetal and placental ultrasound findings, Doppler indices, and cardiotocography; (d) delivery-related characteristics, including delivery outcome and fetal position during labor; (e) maternal perinatal outcomes, including maternal clinical outcomes and complications; (f) neonatal clinical condition at birth, including general condition, Apgar scores, Downes and Ballard scores, anthropometric parameters, hemodynamic indicators, and congenital anomalies; and (g) neonatal laboratory and screening indicators, including newborn eye smear, neonatal screening results, and routine neonatal laboratory tests. Availability of these extended variables is expected to vary across facilities because of differences in clinical indication, local documentation practices, equipment, timing of care, and service disruption during wartime. Accordingly, extended clinical variables will be used primarily for exploratory analyses or sensitivity analyses where data completeness and comparability are sufficient.

### 2.5 Data analysis

Analyses will be conducted in R (RRID: SCR_001905) using longitudinal modeling approaches. The primary analytic aim is to estimate trajectories in prespecified primary mental health outcomes and to test whether trajectories differ by war-related stress exposure. The main exposure will be cumulative war-related stress exposure, derived from predefined ACT-PNM domains, including displacement, family separation, disruption of healthcare access, economic hardship, and direct or indirect exposure to threat. In primary mixed-effects models, war-related stressor exposure will be represented by baseline ACT-PNM-derived stressor burden, and the main test of the study hypothesis will be the interaction between stressor burden and time in predicting trajectories of the primary mental-health outcome family. The primary trajectory contrast will be the war-related stress exposure-by-time interaction across baseline, 3-month, 6-month, and 12-month assessments, with baseline-to-12-month change treated as the principal longitudinal contrast. Secondary aims will examine reproductive intentions, pregnancy planning, reproductive-status transitions, and cohort-specific perinatal outcomes. Exploratory analyses will examine dyadic partner data, sexual attitudes, domestic-violence myth acceptance, and clinician-recorded obstetric, maternal, fetal, and neonatal indicators.

Before analysis, the dataset will be checked for completeness, duplicate submissions, invalid response categories, incompatible units, out-of-range values, inconsistent reproductive status, and temporal inconsistencies between key clinical events. Examples of such inconsistencies include delivery dates preceding pregnancy-related assessments, postpartum status recorded before childbirth, gestational age values outside plausible ranges, or incompatible pregnancy outcome categories. Values will not be reconstructed from other variables unless the derivation rule has been specified in advance.

Clinical-record variables will be incorporated into analyses according to data quality. Core clinical variables will be used to describe the sample, define reproductive timing, and support adjustment or subgroup characterization where relevant. Extended clinical variables will be summarized descriptively and considered for exploratory analyses only when completeness, comparability across sites, and model stability are sufficient. Variables with substantial missingness, inconsistent measurement, or limited cross-site comparability will not be forced into multivariable models.

If a clinical variable is absent, unavailable, not clinically indicated, inaccessible at the site, or cannot be reliably extracted from the local routine record, the corresponding field will be coded as missing or uncertain using predefined coding rules. If a clinician-entered value is missing, implausible, or internally inconsistent, clarification may be requested from the recruiting site using the participant’s study identifier only. The research team will not request copies of clinical charts for verification. The designated site clinician may correct the standardized form entry, confirm that the information is unavailable, or mark the value as uncertain. Corrections will be documented in the study data-management record (87).

Participants will not be excluded from the study solely because some or all clinician-entered clinical variables are unavailable, if eligibility criteria are met and questionnaire data are available. These participants will be retained for analyses based on self-report data and excluded only from analyses requiring the unavailable clinical variables. The extent of missing or uncertain clinician-entered clinical data will be summarized overall, by variable, and by recruitment site. Where substantial site-level differences in clinical-data completeness are observed, sensitivity analyses will be considered, including complete-case analyses for specific clinical variables, exclusion of variables with excessive missingness, or adjustment for recruitment site where appropriate.

Missing data and attrition will be treated as potential sources of bias, particularly because wartime mobility, distress, and changing access to healthcare may affect follow-up participation. Missingness will first be described by variable, wave, reproductive-status group, recruitment site, and partner-participation status. Intermittent missingness, where participants miss one wave but return at a later assessment, will be distinguished from permanent dropout. Reasons for missingness will be coded where available, including withdrawal, non-response, loss of contact, unsafe contact, relocation, inability to complete the questionnaire, non-consent for clinical-data extraction, and unavailable clinical records. Predictors of dropout will be evaluated using multivariable logistic regression models, and standardized mean differences will be used to quantify imbalance between retained and non-retained participants. Models will use full-information maximum likelihood estimation under a missing-at-random assumption (142). Missing covariates will be handled using multiple imputation by chained equations (RRID: SCR_026363), with estimates pooled using Rubin’s rules (143–145). Sensitivity analyses will be used to assess the robustness of primary findings. These will include complete-case analyses, inverse probability-of-censoring weighting based on estimated probability of follow-up retention, and alternative imputation specifications that include baseline distress, war-related exposure, displacement, reproductive status, and recruitment site as predictors of missingness. Where dropout appears related to psychological distress or other outcome-related variables, additional sensitivity analyses will compare primary model estimates before and after applying retention weights and after restricting analyses to participants with at least two completed waves. If results differ materially across these specifications, conclusions will emphasize the direction, size, and robustness of effects rather than a single model estimate. Baseline analyses will first be conducted for the total sample and separately by reproductive-status group. Group differences at baseline will be examined to characterize cohort heterogeneity and to identify variables requiring adjustment in longitudinal models.

Psychometric evaluation of the measures will be undertaken. For newly translated Ukrainian-language measures, psychometric evaluation will be conducted as confirmatory validation analyses; for measures with prior Ukrainian validation evidence, these analyses will be used to support the assessment of measurement quality in the present sample. Psychometric analyses will be conducted separately for each measure within the sample for which the measure is applicable. Internal consistency reliability will be assessed using Cronbach’s alpha and McDonald’s omega. Coefficients of .70 or higher will be considered acceptable, and coefficients of .90 or higher will indicate excellent reliability; coefficients below .70 will be interpreted as indicating limited consistency, whereas coefficients above .95 will be examined for possible item redundancy.

Confirmatory factor analysis (CFA) will be undertaken where the available number of valid responses is sufficient for the complexity of the model. As a rule, CFA will be prioritized when at least 200 valid observations are available for a given measure, with attention also given to the number of items and estimated parameters. If fewer than approximately 100 valid responses are available, or if response categories are sparse, psychometric evaluation will be limited to descriptive item analysis. CFA will be conducted with model specification guided by the factor structures reported for the original or previously validated versions of each instrument. CFA model fit will be evaluated using the comparative fit index (CFI), Tucker–Lewis index (TLI), root mean square error of approximation (RMSEA), and standardized root mean square residual (SRMR). CFI and TLI values of .90 or higher will be interpreted as minimally acceptable and values of approximately .95 or higher as good fit. RMSEA and SRMR values of .08 or lower will be interpreted as acceptable and values of .06 or lower as good fit. These thresholds will be interpreted together with model convergence, factor loadings, residuals, and theoretical coherence.

Item-level analyses will include inspection of response distribution and missingness, floor and ceiling effects, corrected item-total correlations, and score distributions. For measures administered at multiple assessment waves, longitudinal measurement invariance will be evaluated to assess whether the constructs are measured comparably over time. Invariance by respondent characteristics or reproductive status will be examined where item wording, sample size, and model stability permit. Configural, metric, and scalar invariance will be examined sequentially; partial invariance will be considered when full invariance is not supported (146).

The results of these analyses will inform the interpretation of scale scores, between-group comparisons, and longitudinal change estimates. If a translated scale demonstrates poor psychometric performance, substantive analyses using that scale will be reconsidered before interpretation. The research team will inspect item wording, response distributions, factor loadings, residual correlations, reliability estimates, and the effect of individual items on model fit and internal consistency. Depending on the source and extent of the problem, analyses may use theoretically justified subscale scores, conduct sensitivity analyses excluding items, or report the measure descriptively. Measures with inadequate reliability, unstable factor structure, or insufficient evidence of comparability across groups or time will not be used. Any deviations from the planned scoring approach will be reported transparently.

Primary outcomes will be analyzed using mixed-effects growth models with repeated observations nested within participants. Models will include fixed effects for time, war-related stress exposure, and the interaction between them. Time will initially be modeled as categorical; alternative specifications will be compared when empirically justified. Random intercepts will be used to estimate between-person differences in baseline symptom levels, and random slopes for time will be tested to capture heterogeneity in change over time. Model selection will be guided by likelihood-ratio tests, Akaike information criterion, Bayesian information criterion, convergence diagnostics, and variance estimates. The intraclass correlation coefficient will be estimated from unconditional models.

Baseline reproductive status will be included to account for cohort-entry differences between non-pregnant, pregnant, and postpartum participants. In addition, reproductive status assessed at each wave will be modeled as a time-varying covariate when participants transition between reproductive states during follow-up. Where sample size permits, interactions between time, war-related stress exposure, and reproductive status will be examined to assess whether mental-health trajectories differ across reproductive stages. Primary longitudinal analyses will be conducted in the women’s cohort. Mixed-effects growth models will include repeated observations nested within participants and will estimate mental-health trajectories across the four assessment waves. Baseline reproductive status will be included as a cohort-entry characteristic to account for initial differences between non-pregnant, pregnant, and postpartum participants.

Reproductive transitions will also be examined as secondary longitudinal outcomes. Combining groups in this framework is justified because mixed-effects models allow unequal numbers of observations, time-varying covariates, and adjustment for baseline reproductive status. Transition variables will be derived between waves, including, where applicable, becoming pregnant, giving birth, having pregnancy or neonatal loss, entering the postpartum period, remaining in the same reproductive-status group, or other documented reproductive-status changes. Binary transitions will be analyzed using generalized mixed-effects models with a logit link, whereas transitions with more than two categories will be modeled using multinomial or alternative categorical longitudinal models, depending on event frequency and model stability. These analyses will be interpreted as secondary or exploratory if the number of observed transitions is limited.

Values resulting from planned non-administration of a measure (see **Table 3**) will be coded as structurally non-applicable and will be distinguished from missing data due to item nonresponse or loss to follow-up. Structurally non-applicable values will not be imputed or treated as participant-level missingness. Analyses of cohort-specific measures will therefore be restricted to the eligible reproductive-status group or wave, while mixed-effects models will use all available repeated observations for variables applicable across groups.

For postpartum participants, time since childbirth will be incorporated into the analytic framework to account for heterogeneity within the first postpartum year. Postpartum timing will first be described continuously in months since childbirth and, where sample size permits, also examined using clinically meaningful intervals, such as early versus later postpartum periods. In longitudinal models, postpartum timing will be considered as an adjustment variable, a time-varying covariate, or a basis for sensitivity or stratified analyses, depending on the distribution of observations and model stability.

Adjusted models will include selected sociodemographic, obstetric, clinical, and contextual covariates. For time-varying predictors, person-mean centering will be used to distinguish within-person from between-person effects (147). Moderation by risk or protective factors, such as displacement status, prior mental health history, socioeconomic status, social support, and reproductive status, will be tested using interaction terms. Results will be reported as model-based estimates with 95% confidence intervals, p-values, standardized effect estimates, marginal and conditional R², estimated marginal means, contrasts, and predicted trajectory plots. Interpretation will prioritize effect sizes, clinical relevance, and robustness across sensitivity analyses.

To reduce the risk of false-positive findings and model overfitting, the primary analytic model will follow a prespecified hierarchy. The primary longitudinal analysis will focus on WHO-5 trajectories, with baseline war-related stressor burden as the main exposure and the stressor burden-by-time interaction as the principal test of the primary hypothesis, with baseline-to-12-month change treated as the principal longitudinal contrast. Baseline reproductive status will be included to account for cohort-entry differences. Data-driven stepwise selection will not be used for primary inference. For tests conducted across the primary outcome family, Holm-adjusted p-values will be reported alongside unadjusted estimates.

Other mental-health outcomes, reproductive-behavior outcomes, reproductive-status transitions, partner-reported variables, clinical indicators, and contextual measures will be examined as secondary or exploratory analyses according to their role in the analytic framework. These models will be specified with attention to the available sample size, distribution of observations, number of parameters, convergence, and precision of estimates. Where model instability or sparse data are evident, simpler specifications or descriptive presentation will be used. Secondary outcomes will be interpreted as supportive analyses, and exploratory or contextual measures will be interpreted as hypothesis-generating.

### 2.6 Ethics and dissemination

#### 2.6.1 Ethical considerations

The study was registered at ClinicalTrials.gov (identifier: NCT07551934; RRID: SCR_002309). The study protocol was approved by the Bioethics Committee at Taras Shevchenko National University of Kyiv (No. 3/03-04-2026). All participants will provide informed consent before any study procedures. Participation will be voluntary, and refusal to participate, withdrawal from the study, or refusal to provide contact information for follow-up will not affect access to medical care, social support, or any other services in accordance with the Declaration of Helsinki (148). Where clinical information is collected from routine clinical records, this will be done only with the participant’s specific consent.

The study involves sensitive information related to reproductive experiences, perinatal mental health, war-related exposure, suicidality-related symptoms, adverse life experiences, and psychosocial circumstances. To reduce participant burden and potential distress, data collection will use standardized web-based questionnaires with programmed routing logic. Participants will be presented only with questions relevant to their reproductive status and study pathway. Participants may discontinue participation at any time. Information on relevant psychological support services, crisis resources, and contact details for the research team will be provided.

An automated safety-response pathway will be embedded in the web-based questionnaire. The pathway will be activated by predefined thresholds for possible acute risk or clinically elevated distress, including an SCI-2-SF score of ≥ 2, EPDS ≥ 13, GAD-7 ≥ 7, IES-R ≥ 30, or City BiTS ≥ 29. When activated, the questionnaire will immediately display a neutral safety message, information on crisis and psychosocial support services, instructions to contact emergency services or the nearest healthcare facility if immediate danger is present, and options to stop, pause, or continue the questionnaire. After receiving the safety message, participants will retain control over their participation. They may stop the questionnaire, pause and return later, skip any item, or continue if they consider it safe and acceptable. The message will also remind participants that participation is voluntary, and that non-completion or withdrawal will have no consequences for their medical care or access to services. All participants, regardless of whether the safety pathway is activated, will receive mental-health and crisis-support information at the end of the questionnaire.

The automated response will not generate notifications to partners, accompanying persons, or routine clinical staff. Where the participant has provided consent for follow-up contact and has indicated a safe contact method, a trained member of the research team may review the alert according to the approved safety protocol and provide information on available referral pathways. The study team will not provide clinical diagnosis, psychotherapy, emergency monitoring, or real-time crisis intervention as part of the study. Because data are collected through a web-based, pseudonymized questionnaire and responses are not monitored continuously in real time, the study team cannot guarantee individualized emergency response to each risk-related answer. For this reason, the primary safety mechanism is the immediate automated provision of emergency, crisis, and referral information within the survey itself. If a participant independently contacts the research team and reports acute distress, suicidal intent, or risk of self-harm, a trained member of the research team will respond within one day.

The protocol will include a dedicated procedure for the ethical management of reproductive and perinatal loss or severe adverse birth-related events reported during follow-up. Additional event-specific safeguards will be applied if participants report pregnancy loss, stillbirth, neonatal loss, severe obstetric complications, or traumatic birth during follow-up. These events will be handled using a bereavement-sensitive approach. Questionnaire wording and routing will be designed to avoid assumptions about pregnancy continuation, infant survival, or parental experience, and to collect only information necessary for the study aims. Event-related responses will not trigger disclosure to partners, accompanying persons, or clinical staff through the research procedure. Where relevant, participants will be provided with neutral information on psychological, bereavement, crisis, and healthcare support options. These procedures are intended to reduce avoidable distress while allowing participants’ experiences of reproductive or perinatal loss and traumatic birth to be represented in the study if they choose to continue participation.

Several procedures will be used to mitigate potential sources of bias. Selection bias will be mitigated through systematic invitation of eligible participants during predefined recruitment periods. Because the study population will be recruited in healthcare settings and the questionnaire includes sensitive content, additional safeguards will be applied during recruitment. Study invitation will be conducted in a private or semi-private setting whenever feasible, and potential participants will not be asked about trauma exposure, suicidality-related symptoms, intimate-partner circumstances, or other sensitive domains during the initial approach. The invitation will use neutral wording focused on participation in a longitudinal study of reproductive experiences, so that the nature of a participant’s eligibility or interest in the study is not disclosed to accompanying persons. The risk of perceived coercion will be reduced by conducting recruitment through personnel who are not directly responsible for participants’ clinical care, wherever feasible. Potential participants will be able to decline immediately, request more time to consider participation, or complete the consent procedure later using the study link. Refusal to participate will not be recorded in the participant’s clinical record and will not be communicated to clinicians involved in the participant’s care, except where required for basic recruitment monitoring in anonymized or aggregate form. Participants who complete the questionnaire on site will be encouraged to do so privately, using their own device where possible. If privacy cannot be ensured, participants will be offered the option to complete the questionnaire later.

Where partners are invited as a supplementary subgroup, partner recruitment will be handled in a way that preserves the woman’s autonomy and confidentiality. Women’s participation will not require partner involvement, and partners will not be informed about women’s questionnaire responses, consent choices, or follow-up participation. Partner involvement will be treated as optional and conditional on safety, privacy, and voluntariness. Partner data will be collected through a separate consent process and separate questionnaire access, and partners will not receive access to women’s responses, consent decisions, follow-up participation, or safety-related questionnaire outputs. If there is any concern that partner recruitment, dyadic matching, or sharing of a self-generated identification code could increase pressure, surveillance, conflict, or risk to the participant, dyadic linkage will not be pursued and the woman’s individual data will remain eligible for non-dyadic analyses.

Measurement bias will be mitigated through standardized questionnaires, predefined assessment waves, programmed routing logic, and validated instruments where available. Attrition and missing-data bias will be evaluated by documenting retention across waves, examining predictors of dropout, and assessing imbalance between retained and non-retained participants. Missing data will be addressed using appropriate longitudinal estimation methods, multiple imputation for missing covariates where justified, and sensitivity analyses. Confounding will be addressed through adjusted models including theoretically selected sociodemographic, obstetric, clinical, psychological, and contextual covariates.

Additional safeguards will be applied for internally displaced participants and other participants whose safety may be affected by digital follow-up. The study will not collect GPS data, precise shelter locations, or unnecessary address information. Displacement-related information will be collected only at the level required for analysis. Follow-up communications will use neutral wording that does not disclose pregnancy, mental health, trauma, domestic violence, displacement status, or study responses. Participants will be able to choose a safe contact address, change or delete contact information, pause follow-up, or withdraw from further digital contact. Participants will also be reminded to complete the questionnaire only when privacy is available and, where relevant, to avoid using shared devices or unsafe communication channels.

Participant involvement was incorporated during protocol development to improve the relevance, acceptability, and feasibility of the study procedures. Individuals with lived experience of pregnancy planning, pregnancy, childbirth, and motherhood provided input on participant-facing materials and questionnaire content. Their feedback informed the selection and framing of study domains, the clarity of the materials, and the acceptability of study procedures.

#### 2.6.2 Data protection

Data protection and confidentiality will be maintained throughout all stages of the study. All collected data will be processed in accordance with applicable data protection regulations of Ukraine (149). Survey responses, contact information, and clinical or obstetric information will be managed according to the principles of data minimization, restricted access, and pseudonymization. Each participant will be assigned a unique study identifier to enable longitudinal linkage across waves without including direct identifiers in the research dataset. Any transfer of research data outside the primary research team will occur only after removal of direct identifiers and review of re-identification risk.

Access to identifying or linkable data will be restricted to an authorized member of the research team who requires access for follow-up contact and data management. Members of the research team responsible for statistical analysis will work only with pseudonymized datasets. Medical personnel may provide relevant routine clinical or obstetric information through a separate standardized form, but they will not have access to participants’ survey responses. The research team will not access, copy, transfer, store, or centrally review original clinical charts. Original medical records will remain within the participating healthcare institutions, and only predefined chart-derived variables will be entered by authorized healthcare personnel into the study form where the participant has provided specific consent for such data collection. The standardized clinical form will use predefined variable definitions, response options, permitted values, date formats, units of measurement, and missingness reason codes to support comparability across regional hospitals. Where relevant information is absent, inaccessible, not recorded, not clinically indicated, or cannot be reliably extracted from the local record, the corresponding field will be coded as missing or uncertain rather than reconstructed by assumption.

To provide an institutional backend backup for secure dyadic matching, the SGIC will be stored as a linkage variable in the research database together with the respondent-role item, while direct identifying/contact information will be stored separately from questionnaire responses. The restricted identifying file will contain only the minimum information required for follow-up and data management, including the participant’s email address, unique derivative participant code, and secondary pseudonymized study ID. Access to this restricted file will be limited to the person responsible for data management and, where necessary, the authorized research-team member coordinating recruitment and follow-up, according to need-to-know and data-minimization principles. Recruiting clinics may support the initial invitation of eligible participants and, where applicable, enter medical or obstetric indicators into a separate form, but clinic staff will not have access to psychometric questionnaire responses, the full identifying dataset, or the dyadic analytical file.

After completion of participation, withdrawal, or failure to respond after the third follow-up contact, directly identifying information will be deleted according to the study’s data-management procedures (87). If no response is received within 3 months after the last contact attempt, identifying contact data will also be destroyed.

#### 2.6.3 Dissemination of findings

The study findings will be disseminated through peer-reviewed journal publications and presentations at national and international academic conferences in psychology, psychiatry, public health, maternal health, trauma research, reproductive health, and related fields. Where appropriate, results will also be communicated to professional and stakeholder audiences involved in women’s healthcare. The study is expected to produce an empirically grounded integrative model of factors associated with reproductive behavior and perinatal mental health among Ukrainian women during war. It is also expected to generate evidence relevant to the development of perinatal psychological support in humanitarian and post-war recovery contexts.

Both statistically significant and non-significant findings will be reported. Dissemination will be limited to aggregate findings. No information that could identify individual participants, families, healthcare providers, or clinical sites will be disclosed in publications. Following study completion, the documentation necessary for secondary analysis and interpretation will be deposited in the Open Science Framework (RRID: SCR_003238), in accordance with the risk-based open-science plan. Study documentation, the data-management plan, analytic scripts, codebooks, and other non-identifiable materials will be made available, whereas a de-identified analytic dataset may be prepared for sharing only if this can be done without unacceptable re-identification risk. Before any participant-level dataset is deposited or shared, direct identifiers will be removed and indirect identifiers will be reviewed carefully. Variables that may increase re-identification risk, including precise dates, detailed geographic information, recruitment-site identifiers, rare clinical conditions, small subgroup categories, and potentially disclosive combinations will be aggregated or excluded as necessary. If a sufficiently anonymized public-use dataset cannot be prepared safely, access to de-identified individual-level data may be considered only through a controlled-access procedure, subject to reasonable scientific request, ethics and data-protection review, and a data-use agreement.

Data corresponding to measures harmonized with the INTERSECT Study (150) will be shared separately through the secure server provided by City St George’s, University of London, in accordance with the consortium’s governance procedures. Only variables required for consortium-related analyses will be shared through this mechanism. All data shared through this mechanism will be anonymized before transfer.

## 3 Discussion

### 3.1 Study context and implications

Research on reproductive and perinatal mental health in contexts of war and displacement has become increasingly important because armed conflict is not only a direct threat to life, but also a set of prolonged conditions under which pregnancy and childbirth are planned, experienced, and supported (67). Armed conflict has been associated with excess maternal and child mortality (48). At the same time, perinatal outcomes are shaped not only by direct exposure to violence, but also by broader disruptions to living conditions, health services and social support (151–153). Reviews and empirical studies in conflict-affected settings indicate impacts across multiple domains, including access to antenatal and maternity care (151), obstetric and neonatal risk (154, 155), and longer-term consequences for mothers and infants (156, 157). Nevertheless, the available evidence remains heterogeneous across conflicts, populations, and health systems, highlighting the need for context-specific research (48, 158).

Furthermore, armed conflict shapes family formation and reproductive behavior (159). From this perspective, research on women’s reproductive and perinatal health is relevant not only to psychiatry and obstetrics, but also to public health, demography, and service planning. Such research may help identify factors associated with reproductive decisions and contribute to a better understanding of demographic trends in wartime and post-war contexts. It is important to note, however, that altered childbearing intentions and the need for appropriate care once pregnancy occurs are interrelated. Changes in reproductive intentions may affect the timing of pregnancy and care-seeking (160, 161), while inadequate care, insecure living conditions, and elevated distress may further shape reproductive preferences and subsequent decisions (162, 163). War-related distress may influence both reproductive preferences (164) and engagement with health services (165, 166), whereas disrupted care pathways and unstable living conditions may increase psychological vulnerability during pregnancy and after childbirth (19, 167).

Despite the importance of these issues, reproductive and perinatal needs in humanitarian and fragile settings are not yet matched by fully comprehensive and context-sensitive guidance (168). Existing sources cover a wide range of populations, including pregnant and lactating women, women of reproductive age, adolescents, refugees, internally displaced persons, and women and children with disabilities, but important gaps remain in beneficiary coverage, service content, and healthcare strengthening strategies (169, 170). This is directly relevant to research on wartime reproductive behavior and perinatal mental health, as the field requires evidence that can inform not only the description of burden, but also organization of care under fragile conditions. Studies in this area are therefore valuable because they can clarify how reproductive intentions, mental health, and care experiences interact when standard care pathways are disrupted or reconfigured.

In Ukraine, reproductive and perinatal care is delivered in conditions of repeated attacks on healthcare infrastructure, workforce pressure, disrupted referral pathways, and constrained financial resources. Therefore, the findings of this cohort should be used primarily to identify feasible points at which existing services can be made more responsive without substantially increasing the burden on healthcare personnel. In practical terms, the study may inform low-burden and stepped approaches, such as brief psychosocial risk identification within existing reproductive or perinatal encounters, automated digital routing to support resources, clearer triage pathways for severe distress, and flexible follow-up procedures for displaced or mobile participants. Such recommendations should be understood as complementary to routine care rather than as substitutes for specialist mental health services or emergency clinical response.

Against this background, the implications of a study such as the present one extend beyond estimating symptom burden. Longitudinal evidence on pregnancy and childbirth in wartime may help clarify whether war-related stressors are associated with perinatal mental health disorders and with reproductive choices. Such evidence may also help identify where reproductive and perinatal care in fragile settings could be made more responsive. More broadly, studies of this kind may support a more realistic and contextually grounded understanding of reproductive health in war, recognizing that family formation, pregnancy, childbirth, and early parenting unfold within the social, relational, and institutional consequences of war.

### 3.2 Strengths and limitations

A major strength of the present protocol is that it combines several design features that have usually appeared separately in the existing literature. Previous work in war-affected perinatal research has made important contributions, including protocol development for refugee and conflict-affected populations and prospective studies focused on postpartum mental health outcomes. However, the present protocol is distinctive in combining a longitudinal design, repeated assessments, and inclusion of women across multiple reproductive stages. This broader scope may allow the study to examine reproductive behavior and perinatal mental health within a single framework and to characterize change over time.

A further strength is the protocol’s attempt to situate mental health within a wider reproductive and care context. Recent literature in humanitarian and fragile settings has emphasized that reproductive and maternal health guidance remains incomplete in important respects, while integrated approaches to perinatal mental health care are increasingly recognized as necessary but unevenly developed. By linking war-related exposures, mental health measures, reproductive intentions or transitions, and selected routine clinical information within one observational design, the study may improve the contextual and clinical interpretability of its findings compared with work focused on only one domain.

The additional dyadic component is another strength. Although partner involvement is not always feasible in perinatal cohort research, growing evidence suggests that partners’ psychological well-being and couple dynamics may influence reproductive decision-making and perinatal adjustment. Including a mechanism for partner-linked data therefore broadens the conceptual reach of the study and acknowledges that reproductive and perinatal experiences are often relational. At the same time, this should be regarded as an additional opportunity rather than a guaranteed feature of the final dataset, since recruitment and retention of partners are recognized challenges in longitudinal cohort research. Evidence from longitudinal birth cohort research shows that recruitment and retention are influenced by practical burden, time demands, trust, perceived relevance, and the complexity of enrolling not only the pregnant participant but sometimes also the child and partner (171).

Recruitment through healthcare institutions should likewise be viewed in balanced terms. Clinic-based recruitment provides access to women currently navigating reproductive or perinatal care and increases the practical relevance of the study to real-world service settings. It also facilitates linkage, where consent is provided, between self-report and routine clinical data. However, healthcare-based sampling may under-represent women whose contact with services is reduced or interrupted, including those affected by displacement, financial barriers, safety concerns, or disengagement from care. In a wartime setting, this is especially important because uneven access to care may itself form part of the exposure context. The findings will therefore need to be interpreted with awareness that women outside formal care pathways may not be fully represented.

Several methodological limitations inherent to the prospective cohort design should also be acknowledged. Although repeated assessments allow temporal ordering of exposures and outcomes, the observational nature of the study precludes causal inference in the experimental sense. Associations between war-related exposures, reproductive transitions, and mental-health outcomes may be influenced by unmeasured or imperfectly measured confounders, including prior trauma exposure, pre-war mental health, access to care, social support, and changing local security conditions. In addition, reproductive status may change during follow-up, and the timing of these transitions may not be uniform across participants.

More broadly, the protocol should be interpreted in light of the general limitations of ambitious longitudinal observational studies conducted under unstable conditions. Wartime and displacement contexts may affect who is reachable, who is able or willing to participate, and who remains in follow-up. Attrition over 12 months is likely to be a major methodological challenge, particularly in the context of war, mobility, and changing contact circumstances. The protocol addresses this through sample-size planning and flexible follow-up procedures, which is a strength, but loss to follow-up may still reduce statistical precision and introduce bias if discontinuation is associated with distress, instability, or service access. In addition, although the study includes several established instruments, some Ukrainian-language versions are newly translated or still undergoing psychometric evaluation. Interpretation of the eventual findings will therefore need to take measurement performance into account.

## References

[1] Heyne CS Kazmierczak M Souday R Horesh D Lambregtse-van den Berg M Weigl T . Prevalence and risk factors of birth-related posttraumatic stress among parents: A comparative systematic review and meta-analysis. Clin Psychol Rev. (2022) 94:102157. doi: 10.1016/j.cpr.2022.102157 35584590

[2] Dekel S Stuebe C Dishy G . Childbirth induced posttraumatic stress syndrome: a systematic review of prevalence and risk factors. Front Psychol. (2017) 8:560. doi: 10.3389/fpsyg.2017.00560 28443054 PMC5387093

[3] Liu X Wang S Wang G . Prevalence and risk factors of postpartum depression in women: a systematic review and meta-analysis. J Clin Nurs. (2022) 31:2665–77. doi: 10.1111/jocn.16121 34750904

[4] Shorey S Chee CY Ng ED Chan YH San Tam WW Chong YS . Prevalence and incidence of postpartum depression among healthy mothers: A systematic review and meta-analysis. J Psychiatr Res. (2018) 104:235–48. doi: 10.1016/j.jpsychires.2018.08.001 30114665

[5] Roddy Mitchell A Gordon H Lindquist A Walker SP Homer CS Middleton A . Prevalence of perinatal depression in low-and middle-income countries: a systematic review and meta-analysis. JAMA Psychiatry. (2023) 80:425–31. doi: 10.1001/jamapsychiatry.2023.0069 36884232 PMC9996459

[6] Goodman JH Watson GR Stubbs B . Anxiety disorders in postpartum women: a systematic review and meta-analysis. J Affect Disord. (2016) 203:292–331. doi: 10.1016/j.jad.2016.05.033 27317922

[7] Fawcett EJ Fairbrother N Cox ML White IR Fawcett JM . The prevalence of anxiety disorders during pregnancy and the postpartum period: a multivariate Bayesian meta-analysis. J Clin Psychiatry. (2019) 80:18r12527. doi: 10.4088/JCP.18r12527 31347796 PMC6839961

[8] Dennis CL Falah-Hassani K Shiri R . Prevalence of antenatal and postnatal anxiety: systematic review and meta-analysis. Br J Psychiatry. (2017) 210:315–23. doi: 10.1192/bjp.bp.116.187179 28302701

[9] Grote NK Bridge JA Gavin AR Melville JL Iyengar S Katon WJ . A meta-analysis of depression during pregnancy and the risk of preterm birth, low birth weight and intrauterine growth restriction. Arch Gen Psychiatry. (2010) 67:1012–24. doi: 10.1001/archgenpsychiatry.2010.111 20921117 PMC3025772

[10] Grigoriadis S Graves L Peer M Mamisashvili L Tomlinson G Vigod SN . Maternal anxiety during pregnancy and the association with adverse perinatal outcomes: systematic review and meta-analysis. J Clin Psychiatry. (2018) 79:17r12011. doi: 10.4088/JCP.17r12011 30192449

[11] Simonovich SD Nidey NL Gavin AR Piñeros-Leaño M Hsieh WJ Sbrilli MD . Meta-analysis of antenatal depression and adverse birth outcomes in US populations, 2010–20: Study is a meta-analysis of antenatal depression and adverse birth outcomes in the US, 2010–20. Health Aff. (2021) 40:1560–5. doi: 10.1377/hlthaff.2021.00801 34606360

[12] Rogers A Obst S Teague SJ Rossen L Spry EA Macdonald JA . Association between maternal perinatal depression and anxiety and child and adolescent development: a meta-analysis. JAMA Pediatr. (2020) 174:1082–92. doi: 10.1001/jamapediatrics.2020.2910 32926075 PMC7490743

[13] Fan X Wu N Tu Y Zang T Bai J Peng G . Perinatal depression and infant and toddler neurodevelopment: A systematic review and meta-analysis. Neurosci Biobehav Rev. (2024) 159:105579. doi: 10.1016/j.neubiorev.2024.105579 38342472

[14] Bouachba A Gorincour G Charlier P Ville Y . Pregnancy in times of war: what are the fallouts? A review. Fetal Diagn Ther. (2024) 51:559–70. doi: 10.1159/000540508 39047700

[15] Olsen B Forgaard A Nordsletta AH Sommerseth E Røseth I . I shut it out”: expectant mothers’ fear of childbirth after a traumatic birth – a phenomenological study. Int J Qual Stud Health Well-being. (2022) 17:2101209. doi: 10.1080/17482631.2022.2101209 35852421 PMC9302015

[16] Fairbrother N Thordarson DS Stoll K . Fine tuning fear of childbirth: the relationship between Childbirth Fear Questionnaire subscales and demographic and reproductive variables. J Reprod Infant Psychol. (2018) 36:15–29. doi: 10.1080/02646838.2017.1396300 29517300

[17] Krupelnytska L Morozova-Larina O . Perinatal experiences of Ukrainian women at the beginning of the war. J Reprod Infant Psychol. (2025) 43:532–49. doi: 10.1080/02646838.2023.2240827 37485953

[18] Sacchi C . Protection of perinatal mental health during the war in Ukraine. Lancet Reg Health Eur. (2022) 15:100362. doi: 10.1016/j.lanepe.2022.100362 35531497 PMC9072996

[19] Krupelnytska L Vavilova A Yatsenko N Chrzan-Dętkoś M Morozova-Larina O Uka A . War in Ukraine vs. Motherhood: Mental health self-perceptions of relocated pregnant women and new mothers. BMC Preg Childb. (2025) 25:253. doi: 10.1186/s12884-025-07346-0 40057739 PMC11889800

[20] Rock J Yanaşmayan Z . Motherhood on the move: forced migrant women from Ukraine. J Refug Stud. (2025) 38:690–703. doi: 10.1093/jrs/feae084

[21] Tessitore F Gallo M Del Vecchio F Cozzolino M . The feminine and the war: A systematic review on gender-sensitive research during Russia-Ukraine war. Health Care Women Int. (2025) 46:435–52. doi: 10.1080/07399332.2025.2476500 40101110

[22] Milewski N Décieux JP Ette A Bujard M . Gendered flight constellations and family-reunion intentions of female refugees from Ukraine: Evidence from a representative survey in Germany. Cult Pract Eur. (2023) 8:250–63. doi: 10.5771/2566-7742-2023-2-250

[23] Troisi G Tessitore F Celia G Picione RD Margherita G . A bound-less love: long-distance motherhood of Ukrainian women living in Southern Italy. Womens Stud Int Forum. (2024) 105:102939. doi: 10.1016/j.wsif.2024.102939 38826717

[24] Kamenshchikova A Margineau I Munir S Knights F Carter J Requena-Mendez A . Health-care provision for displaced populations arriving from Ukraine. Lancet Infect Dis. (2022) 22:757–9. doi: 10.1016/S1473-3099(22)00225-0 35405089 PMC8993168

[25] Volosovets OP Vyhovska OV Kryvopustov SP Mozyrska OV Yemets OV Volosovets AO . Problems of providing medical care to children of Ukraine as a result of Russian aggression. Childs Health. (2023) 18:157–61. doi: 10.22141/2224-0551.18.3.2023.1578

[26] Haque U Bukhari MH Fiedler N Wang S Korzh O Espinoza J . A comparison of Ukrainian hospital services and functions before and during the Russia-Ukraine war. JAMA Health Forum. (2024) 5:e240901. doi: 10.1001/jamahealthforum.2024.0901 38758566 PMC11102023

[27] United Nations Population Fund . UNFPA Regional Response to Ukraine Emergency Situation Report #26 - 13 February 2025 (2025). Available online at: https://eeca.unfpa.org/en/publications/unfpa-regional-response-Ukraine-emergency-situation-report-26-13-february-2025 (Accessed June 25, 2026).

[28] World Health Organization . Attacks on Ukraine’s Health Care Increased by 20% in 2025 (2026). Available online at: https://www.who.int/news/item/23-02-2026-attacks-on-Ukraine-s-health-care-increased-by-20-in-2025 (Accessed June 25, 2026).

[29] Hebi H Ganem N Wolf MF Lowenstein L Kahalon R Rozenkrantz L . Maternal psychological responses during regional war: A cross-sectional study of fear of childbirth, stress, and intolerance of uncertainty. Biopsychosoc Sci Med. (2026) 88:257–61. doi: 10.1097/PSY.0000000000001444 41198071

[30] McEwen BS Wingfield JC . The concept of allostasis in biology and biomedicine. Horm Behav. (2003) 43:2–15. doi: 10.1016/s0018-506x(02)00024-7 12614627

[31] Shannon M King TL Kennedy HP . Allostasis: a theoretical framework for understanding and evaluating perinatal health outcomes. J Obstet Gynecol Neonatal Nurs. (2007) 36:125–34. doi: 10.1111/j.1552-6909.2007.00126.x 17371513

[32] Premji S . Perinatal distress in women in low- and middle-income countries: Allostatic load as a framework to examine the effect of perinatal distress on preterm birth and infant health. Matern Child Health J. (2014) 18:2393–407. doi: 10.1007/s10995-014-1479-y 24748241 PMC4220111

[33] Stieger S Lewetz D Paschenko S Kurapov A . Examining terror management theory in Ukraine: impact of air-raid alarms and explosions on mental health, somatic symptoms, and well-being. Front Psychiatry. (2023) 14:1244335. doi: 10.3389/fpsyt.2023.1244335 38025457 PMC10644072

[34] Dembitskyi S Sydorov M Moskotina R Zlobina O Kovalska Y . Effect of war and displacement on psychological distress: a study of Ukrainian citizens. Innov Eur J Soc Sci Res. (2025) 38:137–51. doi: 10.1080/13511610.2025.2467836 37339054

[35] Moskalenko L Khanetska N Nizdran O Lutsenko O Pidlypska S . Psychotherapy during war: Working with soldiers and separated families. In: Messias E Peseschkian H , editors. Positive Psychiatry, Psychotherapy and Psychology. Springer, Cham (2025). p. 611–7. doi: 10.1007/978-3-031-94645-5_49

[36] Liashenko O Dluhopolskyi O Koziuk V Zherlitsyn D Dluhopolska T . Societal anxieties and perceived economic vulnerability: How social pessimism shapes financial insecurity across Europe. Societies. (2026) 16:125. doi: 10.3390/soc16040125 30654563

[37] Iborra-Marmolejo I Beneyto-Arrojo MJ Semkiv I Zharova I Esteve-Rodrigo JV Moret-Tatay C . Resilience, mental health, and exposure to death in wartime Ukraine. Eur Psychol. (2026). doi: 10.1027/1016-9040/a000583 37994309

[38] Li Y Dalton VK Lee SJ Rosemberg MA Seng JS . Exploring the validity of allostatic load in pregnant women. Midwifery. (2020) 82:102621. doi: 10.1016/j.midw.2019.102621 31927085

[39] Traylor CS Johnson JD Kimmel MC Manuck TA . Effects of psychological stress on adverse pregnancy outcomes and nonpharmacologic approaches for reduction: an expert review. Am J Obstet Gynecol MFM. (2020) 2:100229. doi: 10.1016/j.ajogmf.2020.100229 32995736 PMC7513755

[40] Costello LA Banker SM Morales S Barber M Hockett C McCormack L . The role of allostatic load in adverse pregnancy outcomes: a multisystem, developmental perspective. Front Glob Womens Health. (2025) 6:1725275. doi: 10.3389/fgwh.2025.1725275 41586415 PMC12824014

[41] Howard LM Oram S Galley H Trevillion K Feder G . Domestic violence and perinatal mental disorders: a systematic review and meta-analysis. PloS Med. (2013) 10:e1001452. doi: 10.1371/journal.pmed.1001452 23723741 PMC3665851

[42] Halim N Beard J Mesic A Patel A Henderson D Hibberd P . Intimate partner violence during pregnancy and perinatal mental disorders in low and lower middle income countries: A systematic review of literature, 1990–2017. Clin Psychol Rev. (2018) 66:117–35. doi: 10.1016/j.cpr.2017.11.004 29198412

[43] Dai X Chu X Qi G Yuan P Zhou Y Xiang H . Worldwide perinatal intimate partner violence prevalence and risk factors for post-traumatic stress disorder in women: a systematic review and meta-analysis. Trauma Violence Abuse. (2024) 25:2363–76. doi: 10.1177/15248380231211950 38001566

[44] Horn R Puffer ES Roesch E Lehmann H . Women’s perceptions of effects of war on intimate partner violence and gender roles in two post-conflict West African countries: consequences and unexpected opportunities. Confl Health. (2014) 8:12. doi: 10.1186/1752-1505-8-12 25104971 PMC4124472

[45] Bourey C Musci RJ Bass JK Glass N Matabaro A Kelly JT . Drivers of men’s use of intimate partner violence in conflict-affected settings: learnings from the Democratic Republic of Congo. Confl Health. (2024) 18:9. doi: 10.1186/s13031-023-00562-5 38254170 PMC10804634

[46] Spangaro J Toole-Anstey C MacPhail CL Rambaldini-Gooding DC Keevers L Garcia-Moreno C . The impact of interventions to reduce risk and incidence of intimate partner violence and sexual violence in conflict and post-conflict states and other humanitarian crises in low and middle income countries: a systematic review. Confl Health. (2021) 15:86. doi: 10.1186/s13031-021-00417-x 34819111 PMC8611888

[47] Liang B Goodman L Tummala-Narra P Weintraub S . A theoretical framework for understanding help‐seeking processes among survivors of intimate partner violence. Am J Community Psychol. (2005) 36:71–84. doi: 10.1007/s10464-005-6233-6 16134045

[48] Jawad M Hone T Vamos EP Cetorelli V Millett C . Implications of armed conflict for maternal and child health: a regression analysis of data from 181 countries for 2000–2019. PloS Med. (2021) 18:e1003810. doi: 10.1371/journal.pmed.1003810 34582455 PMC8478221

[49] Prom MC Denduluri A Philpotts LL Rondon MB Borba CP Gelaye B . A systematic review of interventions that integrate perinatal mental health care into routine maternal care in low-and middle-income countries. Front Psychiatry. (2022) 13:859341. doi: 10.3389/fpsyt.2022.859341 35360136 PMC8964099

[50] Bradford BF Wilson AN Portela A McConville F Fernandez Turienzo C . Midwifery continuity of care: A scoping review of where, how, by whom and for whom? PloS Glob Public Health. (2022) 2:e0000935. doi: 10.1371/journal.pgph.0000935 36962588 PMC10021789

[51] Liebregts J Goodarzi B Valentijn P Downe S Erwich JJ Burchell G . Organising maternal and newborn care in high-income countries: a scoping review of organisational elements and their association with outcomes. BMJ Open. (2025) 15:e107624. doi: 10.1136/bmjopen-2025-107624 41397744 PMC12706211

[52] Choi C Mersky JP Janczewski CE Goyal D . Advancing research on perinatal depression trajectories: evidence from a longitudinal study of low-income women. J Affect Disord. (2022) 301:44–51. doi: 10.1016/j.jad.2022.01.026 34995707

[53] Klapper-Goldstein H Pariente G Wainstock T Dekel S Binyamin Y Battat TL . The association of delivery during a war with the risk for postpartum depression, anxiety and impaired maternal-infant bonding, a prospective cohort study. Arch Gynecol Obstet. (2024) 310:2863–71. doi: 10.1007/s00404-024-07715-8 39367974

[54] Kalfon-Hakhmigari M Handelzalts JE . Women’s postpartum mental health in Israel after terror: childbirth-related trauma following the October 7 attack. J Reprod Infant Psychol. (2026) 11:1. doi: 10.1080/02646838.2026.2625186 41660797

[55] Alfayumi-Zeadna S Bar V Neufeld-Kroszynski G Reuveni I Maugami V Bina R . Perinatal depression during armed conflict in Israel: mental health risks and challenges, a cross-sectional study. Psychiatry Res. (2026) 356:116910. doi: 10.1016/j.psychres.2025.116910 41442956

[56] Abuhaiba S Elijla Y El-Dadah A Nassar O Abu-Sultan M Almasry M . Maternal and neonatal outcomes in Gaza amid armed hostilities in 2025. Int J Gynaecol Obstet. (2026). doi: 10.1002/ijgo.70984 41909974 PMC13505221

[57] Bitan R Magnezi R Reuveni I Tripto A Weinstein O Amikam U . Perinatal outcomes during wartime: a multicenter retrospective cohort study in Israel, 2022–2024. BMC Preg Childb. (2025) 25:1148. doi: 10.1186/s12884-025-08330-4 41162929 PMC12574283

[58] Cevik A Akçay EA Gozuyesil E Vurgec BA Surucu SG . Impact of trauma, support, and control perceptions during childbirth on post-traumatic stress disorder among Syrian immigrant adolescent pregnant women. Midwifery. (2023) 127:103870. doi: 10.1016/j.midw.2023.103870 37931461

[59] Osman S Bakhit MA Osman RM Ahmed AK Adamet KMI . Impact of war on psychological status and quality of life of Sudanese pregnant women. Educ Health. (2025) 38:103–13. doi: 10.62694/efh.2025.290

[60] Callands TA Hylick K Desrosiers A Gilliam SM Taylor EN Hunter JJ . The feasibility and acceptability of Project POWER: a mindfulness-infused, cognitive-behavioral group intervention to address mental and sexual health needs of young pregnant women in Liberia. BMC Preg Childb. (2023) 23:196. doi: 10.1186/s12884-023-05435-6 36941545 PMC10026523

[61] Mirzazada S Padhani ZA Jabeen S Fatima M Rizvi A Ansari U . Impact of conflict on maternal and child health service delivery: a country case study of Afghanistan. Confl Health. (2020) 14:38. doi: 10.1186/s13031-020-00285-x 32536966 PMC7288441

[62] Khan MN Chiumento A Dherani M Bristow K Sikander S Rahman A . Psychological distress and its associations with past events in pregnant women affected by armed conflict in Swat, Pakistan: a cross sectional study. Confl Health. (2015) 9:37. doi: 10.1186/s13031-015-0063-4 26664447 PMC4674905

[63] United Nations Population Fund . UNFPA Regional Response to Ukraine Emergency Situation Report #27 - 5 August 2025 (2025). Available online at: https://eeca.unfpa.org/en/publications/unfpa-regional-response-Ukraine-emergency-situation-report-27-5-august-2025 (Accessed June 25, 2026).

[64] United Nations High Commissioner for Refugees . Ukraine Situation: UNHCR’s 2026 Plans and Financial Requirements (2026). Available online at: https://data.unhcr.org/en/documents/details/120716 (Accessed June 25, 2026).

[65] United Nations Population Fund in Ukraine . Voices From Ukraine Report: Assessment Findings and Recommendations (2025). Available online at: https://Ukraine.unfpa.org/en/publications/voices-Ukraine-pilot (Accessed June 25, 2026).

[66] Verkhovna Rada of Ukraine . Pro Skhvalennia Stratehii Demohrafichnoho Rozvytku Ukrainy Na Period do 2040 Roku. Order of the Cabinet of Ministers of Ukraine; Strategy, Plan, Events on September 30, 2024 № 922-Р (2024). Available online at: https://zakon.rada.gov.ua/go/922-2024-%D1%80 (Accessed June 25, 2026).

[67] Krupelnytska L Morozova-Larina O Yatsenko N Vavilova A . Maternity under fire: the dual vulnerability of Ukrainian women. Arch Womens Ment Health. (2025) 28:283–5. doi: 10.1007/s00737-025-01568-z 39945886

[68] Ryngach NO Vlasyk LY . Changing reproductive plans of the student youth caused by COVID-19 pandemic and the Russian aggression: the potential impact on the birth rate in Ukraine. Stat Ukr. (2025) 109:83–95. doi: 10.31767/su.2(109)2025.02.08

[69] United Nations Population Fund . The Real Fertility Crisis: The Pursuit of Reproductive Agency in a Changing World (2025). Available online at: https://www.unfpa.org/swp2025 (Accessed June 25, 2026).

[70] United Nations Population Fund . Population Matters: Building Demographic Resilience and Modern Statistical System (2026). Available online at: https://Ukraine.unfpa.org/en/topics/population-matters-building-demographic-resilience-and-modern-statistical-system (Accessed June 25, 2026).

[71] Svallfors S Båge K Ekström AM Elimian K Gayawan E Litorp H . Armed conflict, insecurity, and attitudes toward women’s and girls’ reproductive autonomy in Nigeria. Soc Sci Med. (2024) 348:116777. doi: 10.1016/j.socscimed.2024.116777 38569280

[72] Zhao J Qi W Cheng Y Hao R Yuan M Jin H . Influence of perceived stress on fertility intention among women of childbearing age without children: multiple mediating effect of anxiety, family communication and subjective well-being. Reprod Health. (2024) 21:135. doi: 10.1186/s12978-024-01855-5 39294666 PMC11412041

[73] Stevenson K Fellmeth G Edwards S Calvert C Bennett P Campbell OMR . The global burden of perinatal common mental health disorders and substance use among migrant women: a systematic review and meta-analysis. Lancet Public Health. (2023) 8:e203–16. doi: 10.1016/S2468-2667(22)00342-5 36841561

[74] Aria YK Mason-Jones A Keding A . Perinatal mental health among forced migrant women: a scoping review of prevalence and associated factors. Int J Public Health. (2026) 71:1608845. doi: 10.3389/ijph.2026.1608845 41953636 PMC13053488

[75] Moreau C Bonnet C Beuzelin M Blondel B . Pregnancy planning and acceptance and maternal psychological distress during pregnancy: results from the National Perinatal Survey, France, 2016. BMC Preg Childb. (2022) 22:162. doi: 10.1186/s12884-022-04496-3 35227224 PMC8883609

[76] Mahajan R Burza S Bouter LM Sijtsma K Knottnerus A Kleijnen J . Standardized protocol items recommendations for observational studies (SPIROS) for observational study protocol reporting guidelines: protocol for a Delphi study. JMIR Res Protoc. (2020) 9:e17864. doi: 10.2196/17864 33084592 PMC7641775

[77] Vandenbroucke JP Von Elm E Altman DG Gøtzsche PC Mulrow CD Pocock SJ . Strengthening the Reporting of Observational Studies in Epidemiology (STROBE): explanation and elaboration. PloS Med. (2007) 4:1628–55. doi: 10.1371/journal.pmed.0040297 17941715 PMC2020496

[78] Bornstein M Gemmill A Norris AH Huber-Krum S Gipson JD . Pregnancy and pregnancy intention after experiencing infertility: a longitudinal study of women in Malawi. PloS Glob Public Health. (2023) 3:e0001646. doi: 10.1371/journal.pgph.0001646 37963107 PMC10645290

[79] Speizer IS Escamilla V Lance PM Guilkey DK . Longitudinal examination of changing fertility intentions and behaviors over a four-year period in urban Senegal. Reprod Health. (2020) 17:38. doi: 10.1186/s12978-020-0893-4 32183890 PMC7077111

[80] Iddi S Donohue MC . Power and sample size for longitudinal models in R—the longpower package and shiny app. R J. (2022) 14:264. doi: 10.32614/RJ-2022-022 41552781 PMC12810882

[81] Harrall KK Muller KE Starling AP Dabelea D Barton KE Adgateet JL . Power and sample size analysis for longitudinal mixed models of health in populations exposed to environmental contaminants: a tutorial. BMC Med Res Methodol. (2023) 23:12. doi: 10.1186/s12874-022-01819-y 36635621 PMC9835314

[82] Mor S Sela Y Lev-Ari S . Postpartum mothers’ mental health in a conflict-affected region: a cross-sectional study of emotion regulation and social support. J Clin Med. (2025) 14:1244. doi: 10.3390/jcm14041244 40004775 PMC11856334

[83] Qandil S Jabr S Wagler S Collin SM . Postpartum depression in the Occupied Palestinian Territory: a longitudinal study in Bethlehem. BMC Preg Childb. (2016) 16:375. doi: 10.1186/s12884-016-1155-x 27887649 PMC5124263

[84] Damtew SA Shiferaw S Demissie TD Addissie A Sisay TA Amogne A . Postpartum emotional fertility intentions in Ethiopia: an insight and correlates from a national women and newborns cohort study. BMC Public Health. (2026) 26:37. doi: 10.1186/s12889-025-25311-6 41316181 PMC12764142

[85] Silove D Bryant R Rees S Tam NDJ Dadds M Eapen V . Longitudinal path analysis of depressive symptoms and functioning among women of child-rearing age in postconflict Timor-Leste. BMJ Glob Health. (2020) 5:e002039. doi: 10.1136/bmjgh-2019-002039 32337078 PMC7170425

[86] Rodríguez-Muñoz MF Chrzan-Dętkoś M Uka A García-López HS Krupelnytska L Morozova-Larina O . The impact of the war in Ukraine on the perinatal period: Perinatal Mental Health for Refugee Women (PMH-RW) protocol. Front Psychol. (2023) 14:1152478. doi: 10.3389/fpsyg.2023.1152478 36993880 PMC10042139

[87] Yatsenko NV Krupelnytska L Baratiuk A Kazakov S Morozova-Larina O Molotokas A . Integrative model of factors influencing reproductive behavior and perinatal mental health of women amidst war. In: Open Science Framework. doi: 10.17605/OSF.IO/MV49P

[88] Harani I Ben-Porat A . Intimate partner violence: a dyadic examination of self-differentiation and responsive caregiving. J Marriage Fam. (2026) 88:36–49. doi: 10.1111/jomf.13077 40046247

[89] Heidari S Babor TF De Castro P Tort S Curno M . Sex and gender equity in research: rationale for the SAGER guidelines and recommended use. Res Integr Peer Rev. (2016) 1:2. doi: 10.1186/s41073-016-0007-6 29451543 PMC5793986

[90] Brislin RW . Back-translation for cross-cultural research. J Cross Cult Psychol. (1970) 1:185–216. doi: 10.1177/135910457000100301

[91] Cruchinho P López-Franco MD Capelas ML Almeida S Bennett PM da Silva MM . Translation, cross-cultural adaptation, and validation of measurement instruments: a practical guideline for novice researchers. J Multidiscip Healthc. (2024) 17:2701–28. doi: 10.2147/JMDH.S419714 38840704 PMC11151507

[92] Weiss DS Marmar CR . The impact of event scale – revised. In: Wilson JP Keane TM , editors. Assessing Psychological Trauma and Ptsd: A Practitioner’s Handbook. Guilford Press, New York (1997). p. 399–411.

[93] Moreira Ramiro AC Côrtes Ribeiro C Leles Vieira de Souza B dos Passos FS . Assessment of the psychological impact of the COVID-19 pandemic on pregnant women. J Matern Fetal Neonatal Med. (2022) 35:6461–5. doi: 10.1080/14767058.2021.1915976 33899672

[94] Ahmed AM Mostafa N Dwedar LM . Psychological distress associated with COVID-19 pandemic among pregnant women: a comparative study. Egypt J Health Care. (2020) 11:645–56. doi: 10.21608/ejhc.2020.170295

[95] Ostacoli L Cosma S Bevilacqua F Berchialla P Bovetti M Carosso AR . Psychosocial factors associated with postpartum psychological distress during the COVID-19 pandemic: a cross-sectional study. BMC Preg Childb. (2020) 20:703. doi: 10.1186/s12884-020-03399-5 33208115 PMC7671935

[96] Şimsek-Çetinkaya Ş Şimsek F . The interplay of post-traumatic stress symptoms, psychological resilience, and mother–infant attachment in predicting postpartum depression after earthquakes. Dev Psychobiol. (2026) 68:e70096. doi: 10.1002/dev.70096 41332171

[97] Krupelnytska L Yatsenko N Keller V Morozova-Larina O . The Impact of Events Scale-Revised (IES-R): validation of the Ukrainian version. Compr Psychiatry. (2025) 139:152593. doi: 10.1016/j.comppsych.2025.152593 40168846

[98] Felitti VJ Anda RF Nordenberg D Williamson DF Spitz AM Edwards V . Relationship of childhood abuse and household dysfunction to many of the leading causes of death in adults: the Adverse Childhood Experiences (ACE) Study. Am J Prev Med. (1998) 14:245–58. doi: 10.1016/S0749-3797(98)00017-8 9635069

[99] Lotzin A Krupelnytska L Morozova-Larina O . Mental health and academic performance of university students during the war in Ukraine. In: Open Science Framework (2023). doi: 10.17605/OSF.IO/FPYK5

[100] World Health Organization Regional Office for Europe . Wellbeing Measures in Primary Health Care/the Depcare Project: Report on a WHO Meeting, Stockholm, Sweden, 12–13 February 1998. Copenhagen: World Health Organization Regional Office for Europe (1998). Available online at: https://iris.who.int/handle/10665/349766 (Accessed June 25, 2026).

[101] Bech P Olsen LR Kjoller M Rasmussen NK . Measuring well-being rather than the absence of distress symptoms: a comparison of the SF-36 mental health subscale and the WHO-five well-being scale. Int J Methods Psychiatr Res. (2003) 12:85–91. doi: 10.1002/mpr.145 12830302 PMC6878541

[102] Topp CW Østergaard SD Søndergaard S Bech P . The WHO-5 well-being index: a systematic review of the literature. Psychother Psychosom. (2015) 84:167–76. doi: 10.1159/000376585 25831962

[103] Christiansen CH Christensen KB Høgh S Renault KM Emborg MS Frokjaer VG . Measuring psychological well-being in a Danish pregnancy cohort using the self-reported WHO-5 index. BMC Psychol. (2025) 13:41. doi: 10.1186/s40359-025-02343-6 39815351 PMC11737152

[104] Cox JL Holden JM Sagovsky R . Detection of postnatal depression: development of the 10-item Edinburgh postnatal depression scale. Br J Psychiatry. (1987) 150:782–6. doi: 10.1192/bjp.150.6.782 3651732

[105] Levis B Negeri Z Sun Y Benedetti A Thombs BD DEPRESsion Screening Data (DEPRESSD) EPDS Group . Accuracy of the Edinburgh postnatal depression scale (EPDS) for screening to detect major depression among pregnant and postpartum women: systematic review and meta-analysis of individual participant data. BMJ. (2020) 371:m4022. doi: 10.1136/bmj.m4022 33177069 PMC7656313

[106] Chrzan-Dętkoś M Liakea I Murawska N Costa R Uka A Krupelnytska L . Validation of Edinburgh postnatal depression scale (EPDS) in perinatal women under war conditions in Ukraine. Clin Health. (2026) 37:e260717. doi: 10.5093/clh2026a6

[107] Lautarescu A Victor S Lau-Zhu A Counsell SJ Edwards AD Craig MC . The factor structure of the Edinburgh postnatal depression scale among perinatal high-risk and community samples in London. Arch Womens Ment Health. (2022) 25:157–69. doi: 10.1007/s00737-021-01153-0 34244862 PMC8784492

[108] Ayers S Wright DB Thornton A . Development of a measure of postpartum PTSD: the City Birth Trauma Scale. Front Psychiatry. (2018) 9:409. doi: 10.3389/fpsyt.2018.00409 30279664 PMC6153962

[109] American Psychiatric Association . Diagnostic and Statistical Manual of Mental Disorders. 5th ed, text revision (DSM-5-TR. Washington, DC: American Psychiatric Association (2022). doi: 10.1176/appi.books.9780890425787

[110] Osório FL Rossini Darwin AC Bombonetti EA Ayers S . Posttraumatic stress following childbirth: psychometric properties of the Brazilian version of the City Birth Trauma Scale. J Psychosom Obstet Gynaecol. (2022) 43:374–83. doi: 10.1080/0167482X.2021.1977278 34570669

[111] Chrzan-Dętkoś M Murawska N Krupelnytska L Moreira H Rodríguez-Muñoz MF Costa R . Assessment of childbirth-related PTSD: psychometric properties of the Ukrainian version of the City Birth Trauma Scale. Psychol Trauma. (2026) 18:57–69. doi: 10.1037/tra0001979 41505302

[112] Spitzer RL Kroenke K Williams JBW Löwe B . A brief measure for assessing generalized anxiety disorder: the GAD-7. Arch Intern Med. (2006) 166:1092–7. doi: 10.1001/archinte.166.10.1092 16717171

[113] Gong Y Zhou H Zhang Y Zhu X Wang X Shen B . Validation of the 7-item generalized anxiety disorder scale (GAD-7) as a screening tool for anxiety among pregnant Chinese women. J Affect Disord. (2021) 282:98–103. doi: 10.1016/j.jad.2020.12.129 33401129

[114] Zhong QY Gelaye B Zaslavsky AM Fann JR Rondon MB Sánchez SE . Diagnostic validity of the generalized anxiety disorder-7 (GAD-7) among pregnant women. PloS One. (2015) 10:e0125096. doi: 10.1371/journal.pone.0125096 25915929 PMC4411061

[115] Aleksina N Gerasimenko O Lavrynenko D Savchenko O . Ukrainian adaptation of the generalized anxiety disorder scale (GAD-7): diagnostic experience in the state of martial law. Insight Psychol Dimens Soc. (2024) 11:77–103. doi: 10.32999/2663-970X/2024-11-5 41354707

[116] De Luca GP Parghi N El Hayek R Bloch-Elkouby S Peterkin D Wolfe A . Machine learning approach for the development of a crucial tool in suicide prevention: the Suicide Crisis Inventory-2 (SCI-2) short form. PloS One. (2024) 19:e0299048. doi: 10.1371/journal.pone.0299048 38728274 PMC11086905

[117] Yatsenko NV Somova OO Svirin YV . Psychometric properties of the Ukrainian version of the Suicide Crisis Inventory-2 short form (SCI-2-SF): a sample of Ukrainian Air Assault Forces combatants. Nauk Perspect. (2024) 8:979–93. doi: 10.52058/2708-7530-2024-8(50)-979-993 34076676

[118] Rocca CH Ralph L Wilson M Gould H Foster DG . Psychometric evaluation of an instrument to measure prospective pregnancy preferences: the Desire to Avoid Pregnancy Scale. Med Care. (2019) 57:152–8. doi: 10.1097/MLR.0000000000001048 30550399 PMC6331264

[119] Hall J Barrett G Rocca C . Evaluation of the Desire to Avoid Pregnancy Scale in the UK: a psychometric analysis including predictive validity. BMJ Open. (2022) 12:e060287. doi: 10.1136/bmjopen-2021-060287 35879004 PMC9328097

[120] Hall JA Barrett G Stephenson JM Edelman NL Rocca C . Desire to Avoid Pregnancy Scale: clinical considerations and comparison with other questions about pregnancy preferences. BMJ Sex Reprod Health. (2023) 49:167–75. doi: 10.1136/bmjsrh-2022-201750 36717217 PMC10359540

[121] Barrett G Smith SC Wellings K . Conceptualisation, development, and evaluation of a measure of unplanned pregnancy. J Epidemiol Community Health. (2004) 58:426–33. doi: 10.1136/jech.2003.014787 15082745 PMC1732751

[122] Smorti M Christiansen P Barrett G Hall JA Ionio C Ciuffo G . Psychometric evaluation of the validity and reliability of the Italian version of the London Measure of Unplanned Pregnancy amongst postnatal women. Healthc (Basel). (2025) 13:2052. doi: 10.3390/healthcare13162052 40868668 PMC12386161

[123] Brima N Samba TT Yamba A Barrett G Stephenson J Hall J . Evaluation of the krio language version of the London measure of unplanned pregnancy in Western Area, Sierra Leone. Afr J Reprod Health. (2019) 23:81–91. doi: 10.29063/ajrh2019/v23i4.10 32227743

[124] Altiparmak S Yilmaz AN Aksoy Derya Y . The Turkish validity and reliability study of the London measure of unplanned pregnancy. J Obstet Gynaecol Res. (2021) 47:1362–70. doi: 10.1111/jog.14678 33496061

[125] Hall JA Stewart C Stoneman B Bicknell T Lovell H Duncan H . Implementation of the London Measure of Unplanned Pregnancy in routine antenatal care: a mixed-methods evaluation in three London NHS Trusts. Eur J Midwifery. (2024) 8:10.18332. doi: 10.18332/ejm/188118 38832251 PMC11145722

[126] Webb R Smith AM Ayers S Wright DB Thornton A . Development and validation of a measure of birth-related PTSD for fathers and birth partners: the City Birth Trauma Scale (partner version). Front Psychol. (2021) 12:596779. doi: 10.3389/fpsyg.2021.596779 33746826 PMC7966709

[127] Sandoz V Lacroix A Jubin M Hingray C El Hage W Horsch A . The latent factor structure and assessment of childbirth-related PTSD in fathers: psychometric characteristics of the City Birth Trauma Scale—French version (partner version). Psychol Trauma. (2023) 15:1145–54. doi: 10.1037/tra0001407 36689375

[128] Osório FL Ayers S Gonçalves F Rocha JC . City birth trauma scale-updates to the Portuguese version. Arch Clin Psychiatry (São Paulo). (2021) 48:250. doi: 10.15761/0101-60830000000316

[129] Haga SM Bergunde L Seefeld L Ayers S Eberhard‐Gran M Garthus‐Niegel S . Validation of the City Birth Trauma Scale in a sample of Norwegian mothers. Acta Obstet Gynecol Scand. (2026) 105:508–18. doi: 10.1111/aogs.70149 41588651 PMC12942060

[130] Martin CJ Martin CR . Development and psychometric properties of the Birth Satisfaction Scale-Revised (BSS-R). Midwifery. (2014) 30:610–9. doi: 10.1016/j.midw.2013.10.006 24252712

[131] Martin CR Hollins Martin CJ Burduli E Barbosa-Leiker C Donovan-Batson C Fleming SE . The Birth Satisfaction Scale–Revised (BSS-R): should the subscale scores or the total score be used? J Reprod Infant Psychol. (2018) 36:530–5. doi: 10.1080/02646838.2018.1490498 30058370

[132] Nakić Radoš S Matijaš M Brekalo M Hollins Martin CJ Martin CR . Further validation of the birth satisfaction scale-revised: factor structure, validity, and reliability. Curr Psychol. (2023) 42:13693–702. doi: 10.1007/s12144-021-02688-2 30311153

[133] Jefford E Hollins Martin CJ Martin CR . Development and validation of the Australian version of the Birth Satisfaction Scale-Revised (BSS-R). J Reprod Infant Psychol. (2018) 36:42–58. doi: 10.1080/02646838.2017.1396302 29517299

[134] Göncü Serhatlıoğlu S Karahan N Hollins Martin CJ Martin CR . Construct and content validity of the Turkish Birth Satisfaction Scale–Revised (T-BSS-R). J Reprod Infant Psychol. (2018) 36:235–45. doi: 10.1080/02646838.2018.1443322 29553295

[135] Jawed-Wessel S Herbenick D Schick V Fortenberry JD Cattelona G Reece M . Development and validation of the maternal and partner sex during pregnancy scales. J Sex Marital Ther. (2016) 42:681–701. doi: 10.1080/0092623X.2015.1113587 26684371

[136] Tavares IM Heiman JR Rosen NO Nobre PJ . Validation of the maternal and partner sex during pregnancy scales (MSP/PSP) in Portugal: assessing dyadic interdependence and associations with sexual behaviors. J Sexual Med. (2021) 18:789–99. doi: 10.1016/j.jsxm.2021.01.184 33766522

[137] Ashrafinia F Jawed‐Wessel S Haji‐Maghsoudi S Heydari O Shojaee Baghini F . Investigation of the validity and reliability of the maternal sex during pregnancy scale in Muslim women. Int J Gynecol Obstetr. (2025) 170:338–44. doi: 10.1002/ijgo.16193 39945217

[138] Patrick ME Maggs JL Cooper ML Lee CM . Measurement of motivations for and against sexual behavior. Assessment. (2011) 18:502–16. doi: 10.1177/1073191110372298 20581394 PMC2974960

[139] Peters J . Measuring myths about domestic violence: development and initial validation of the Domestic Violence Myth Acceptance Scale. J Aggress Maltreat Trauma. (2008) 16:1–21. doi: 10.1080/10926770801917780 37339054

[140] Lelaurain S Fonte D Graziani P Monaco GL . French validation of the domestic violence myth acceptance scale (DVMAS). Affilia. (2019) 34:237–58. doi: 10.1177/0886109918806273

[141] Giger JC Gonçalves G Almeida AS . Adaptation of the domestic violence myth acceptance scale to Portuguese and tests of its convergent, divergent, and predictive validities. Violence Against Women. (2017) 23:1790–810. doi: 10.1177/1077801216666724 27758897

[142] Cham H Reshetnyak E Rosenfeld B Breitbart W . Full information maximum likelihood estimation for latent variable interactions with incomplete indicators. Multivariate Behav Res. (2017) 52:12–30. doi: 10.1080/00273171.2016.1245600 27834491 PMC5489914

[143] Resche-Rigon M White IR . Multiple imputation by chained equations for systematically and sporadically missing multilevel data. Stat Methods Med Res. (2018) 27:1634–49. doi: 10.1177/0962280216666564 27647809 PMC5496677

[144] Austin PC White IR Lee DS van Buuren S . Missing data in clinical research: a tutorial on multiple imputation. Can J Cardiol. (2021) 37:1322–31. doi: 10.1016/j.cjca.2020.11.010 33276049 PMC8499698

[145] Rubin DB . Inference and missing data. In: Biometrika, vol. 63. Oxford: Oxford University Press. (1976). p. 581–92. Available online at: https://www.jstor.org/stable/2335739 (Accessed June 25, 2026).

[146] Putnick DL Bornstein MH . Measurement invariance conventions and reporting: the state of the art and future directions for psychological research. Dev Rev. (2016) 41:71–90. doi: 10.1016/j.dr.2016.06.004 27942093 PMC5145197

[147] Curran PJ Bauer DJ . The disaggregation of within-person and between-person effects in longitudinal models of change. Annu Rev Psychol. (2011) 62:583–619. doi: 10.1146/annurev.psych.093008.100356 19575624 PMC3059070

[148] World Medical Association . World Medical Association Declaration of Helsinki: ethical principles for medical research involving human participants. JAMA. (2025) 333:71–4. doi: 10.1001/jama.2024.21972 39425955

[149] Verkhovna Rada of Ukraine . On protection of personal data : law of Ukraine on june 1, 2010 № 2297-VI (2025). Available online at: https://zakon.rada.gov.ua/go/2297-17 (Accessed June 25, 2026).

[150] Ayers S Horsch A Garthus-Niegel S Nieuwenhuijze M Bogaerts A Hartmann K . Traumatic birth and childbirth-related post-traumatic stress disorder: international expert consensus recommendations for practice, policy, and research. Women Birth. (2024) 37:362–7. doi: 10.1016/j.wombi.2023.11.006 38071102

[151] Alibhai KM Ziegler BR Meddings L Batung E Luginaah I . Factors impacting antenatal care utilization: a systematic review of 37 fragile and conflict-affected situations. Confl Health. (2022) 16:33. doi: 10.1186/s13031-022-00459-9 35690840 PMC9188725

[152] Boukari Y Kadir A Waterston T Jarrett P Harkensee C Dexter E . Gaza, armed conflict and child health. BMJ Paediatr Open. (2024) 8:e002407. doi: 10.1136/bmjpo-2023-002407 38350977 PMC10868171

[153] Garry S Checchi F . Armed conflict and public health: into the 21st century. J Public Health (Oxf). (2020) 42:e287–98. doi: 10.1093/pubmed/fdz095 31822891

[154] Riquelme‐Gallego B Ramos‐Soberbio L Leno‐Duran E Martínez‐Vázquez S Caparros‐Gonzalez RA . Adverse fetal and neonatal impact of war conflicts during pregnancy: a systematic review. IUBMB Life. (2025) 77:e70006. doi: 10.1002/iub.70006 39981676

[155] Keasley J Blickwedel J Quenby S . Adverse effects of exposure to armed conflict on pregnancy: a systematic review. BMJ Glob Health. (2017) 2:e000377. doi: 10.1136/bmjgh-2017-000377 29333283 PMC5706483

[156] Kadir A Shenoda S Goldhagen J . Effects of armed conflict on child health and development: a systematic review. PloS One. (2019) 14:e0210071. doi: 10.1371/journal.pone.0210071 30650095 PMC6334973

[157] Qouta SR Vänskä M Diab SY Punamäki RL . War trauma and infant motor, cognitive, and socioemotional development: maternal mental health and dyadic interaction as explanatory processes. Infant Behav Dev. (2021) 63:101532. doi: 10.1016/j.infbeh.2021.101532 33588286

[158] Urdal H Che CP . War and gender inequalities in health: the impact of armed conflict on fertility and maternal mortality. Int Interact. (2013) 39:489–510. doi: 10.1080/03050629.2013.805133 37339054

[159] Kurylo I Aksyonova S Krimer B . Fertility and childbearing plans in Ukraine: war and post-wartime expectations. Calitatea Vieții. (2025) 36:241–70. doi: 10.46841/RCV.2025.04.01

[160] Mekonnen BD Vasilevski V Bali AG Sweet L . Effect of pregnancy intention on completion of maternity continuum of care in Sub-Saharan Africa: systematic review and meta-analysis. BMC Preg Childb. (2024) 24:802. doi: 10.1186/s12884-024-06998-8 39609727 PMC11603981

[161] Khan MN Harris ML Shifti DM Laar AS Loxton D . Effects of unintended pregnancy on maternal healthcare services utilization in low-and lower-middle-income countries: systematic review and meta-analysis. Int J Public Health. (2019) 64:743–54. doi: 10.1007/s00038-019-01238-9 31041453

[162] Shorey S Yang YY Ang E . The impact of negative childbirth experience on future reproductive decisions: a quantitative systematic review. J Adv Nurs. (2018) 74:1236–44. doi: 10.1111/jan.13534 29394456

[163] Joensuu JM Saarijärvi H Rouhe H Gissler M Ulander VM Heinonen S . Effect of the maternal childbirth experience on a subsequent birth: a retrospective 7-year cohort study of primiparas in Finland. BMJ Open. (2023) 13:e069918. doi: 10.1136/bmjopen-2022-069918 36894202 PMC10008220

[164] Krakhmalova K Kloc‐Nowak W . The case of pre‐24 February 2022 Ukrainian migrants and war refugees in Poland: how war affects fertility beliefs and intentions. Popul Space Place. (2025) 31:e70072. doi: 10.1002/psp.70072 41531421

[165] Chrzan-Dętkoś M Murawska NE . We are in this together–Polish midwives’ reflections on perinatal care for Ukrainian women after the outbreak of war. Health Psychol Rep. (2023) 11:177–87. doi: 10.5114/hpr/161996 38084265 PMC10670767

[166] Namasivayam A González PA Delgado RC Chi PC . The effect of armed conflict on the utilization of maternal health services in Uganda: a population-based study. PloS Curr. (2017) 9:ecurrents.dis.557b987d6519d8c7c96f2006ed3c271a. doi: 10.1371/currents.dis.557b987d6519d8c7c96f2006ed3c271a 29188138 PMC5693797

[167] Rodríguez-Muñoz MF Chrzan-Dętkoś M Uka A Fellmeth G . Wars as a source of maternal stress during pregnancy and postpartum. In: Caparros-Gonzalez RA , editor. Maternal Stress During Pregnancy and Postpartum. Springer Nature Switzerland, Cham (2025). p. 103–24. doi: 10.1007/978-3-032-09245-8_6

[168] Rodriguez-Muñoz MF Chrzan-Dętkoś M Uka A Garcia-López HS Bina R Le HN . A narrative review on emerging issues about war-related trauma in perinatal women: good practice for assessment, prevention, and treatment. Arch Womens Ment Health. (2025) 28:201–17. doi: 10.1007/s00737-024-01537-y 39638974

[169] Brackstone K Denny J Hansen AC Trautwein M Badri E Cheran A . Civilian shelter guidance in armed conflict zones: a qualitative study of international humanitarian practitioners. Disast Med Public Health Prep. (2026) 20:e67. doi: 10.1017/dmp.2026.10340 41940523

[170] Wainaina GM Kaura D Jordan P . A systematic review on continuity of care for effective coordination in maternal and neonatal health continuum: experiences of skilled birth attendants. Int J Afr Nurs Sci. (2024) 21:100776. doi: 10.1016/j.ijans.2024.100776 38826717

[171] McKiever M Frey H Costantine MM . Challenges in conducting clinical research studies in pregnant women. J Pharmacokinet Pharmacodyn. (2020) 47:287–93. doi: 10.1007/s10928-020-09687-z 32306165 PMC8237366

